# Programmed activation of cancer cell apoptosis: A tumor-targeted phototherapeutic topoisomerase I inhibitor

**DOI:** 10.1038/srep29018

**Published:** 2016-07-04

**Authors:** Weon Sup Shin, Jiyou Han, Rajesh Kumar, Gyung Gyu Lee, Jonathan L. Sessler, Jong-Hoon Kim, Jong Seung Kim

**Affiliations:** 1 Department of Chemistry, Korea University, Seoul 136-701, Korea; 2 Department of Biotechnology, Laboratory of Stem Cells and Tissue Regeneration, College of Life Sciences & Biotechnology, Korea University, Seoul 136-713, Republic of Korea; 3 Department of Chemistry, University of Texas at Austin, Austin, TX 78712-1224, USA

## Abstract

We report here a tumor-targeting masked phototherapeutic agent 1 (**PT-1**). This system contains SN-38—a prodrug of the topoisomerase I inhibitor irinotecan. Topoisomerase I is a vital enzyme that controls DNA topology during replication, transcription, and recombination. An elevated level of topoisomerase I is found in many carcinomas, making it an attractive target for the development of effective anticancer drugs. In addition, **PT-1** contains both a photo-triggered moiety (nitrovanillin) and a cancer targeting unit (biotin). Upon light activation in cancer cells, **PT-1** interferes with DNA re-ligation, diminishes the expression of topoisomerase I, and enhances the expression of inter alia mitochondrial apoptotic genes, death receptors, and caspase enzymes, inducing DNA damage and eventually leading to apoptosis. *In vitro* and *in vivo* studies showed significant inhibition of cancer growth and the hybrid system **PT-1** thus shows promise as a programmed photo-therapeutic (“phototheranostic”).

Considerable interest has been focused lately on the development of so-called targeted therapeutics. In the case of cancer drug development, this approach is attractive in that it may allow dose-limiting systemic toxicities to be overcome via increased delivery of an active agent to a neoplastic site. For this strategy to be most effective, we believe it is necessary not only to effect delivery, but also controlled release. Appealing also would be an ability to monitor uptake and localization through optical or spectroscopic means. Examples of cancer-targeted, site-activated, and readily visualized antitumor agents are rare. To our knowledge none involving photo-activated release of an active agent has been reported to date. Here we report such a “phototheranostic” system; it is based on inhibition of topoisomerase I.

Topoisomerase I is a critical enzyme that helps control DNA topology, for example, during replication, transcription, repair, and recombination^1^. Human topoisomerase I reversibly mends single strand disruptions and relaxation in DNA, and is a crucial step in DNA replication in healthy cells^2^. An elevated level of topoisomerase I is found in many carcinomas. This has made DNA topoisomerase I a good target for the development of anticancer drugs. Two pentacyclic-quinolone-based analogues of camptothecin, topotecan and irinotecan, that function as topoisomerase I inhibitors, have been approved by the US Food and Drug Administration for the treatment of cancer^3^,^4^. Despite their clinical success, these two drugs suffer from limitations, including low target specificity to cancer cells, adverse effects on healthy cells (e.g., hematological toxicity, liver dysfunction, and anemia), drug resistance due to multidrug resistance transporters (MDRs), and a less-than-ideal therapeutic index. In order to overcome these limitations, a number of topoisomerase I inhibitor prodrugs, activated by certain tumor characteristics, such as reactive oxygen species, pH^5^,^6^, enzymes^7^,^8^, and intracellular thiols^9^,^10^,^11^, have been developed. Unfortunately, most of these agents display toxicity towards healthy cells. We were thus attracted to photo-activation^12^.

Irradiation with light provides the promise of activation with high temporal and spatial resolution and with potentially minimal toxicity^13^. To date, light-activated compounds have been widely studied in a number of chemical and biological contexts^13^,^14^,^15^,^16^,^17^. Furthermore, light-sensitive nanoparticles, such as polymeric up-conversion nanoparticles have been utilized in various practical applications^18^,^19^,^20^,^21^. In the case of cancer treatment, the use of a light-activated prodrug may allow for more precise control of drug release at the tumor site, along with the potential for both modulating the therapeutic activity. To our knowledge, however, the use of light-activated compounds for cancer drug release and the concurrent monitoring of targeted drug delivery has not been explored.

As detailed below, we have now constructed a tumor-targeting masked phototherapeutic agent 1 (**PT-1**) that contains 7-ethyl-10-hydroxycamptothecin (SN-38)—a prodrug of irinotecan—and nitrovanillin as a phototriggered moiety (Fig. 1a). This phototheranostic consists of three moieties. The first is a biotin unit, a cancer-targeting unit that draws the antitumor agent to cancer cells selectively^22^. The second component is a light-activated unit, *o*-nitrobenzyl (nitrovanillin), which provides a fluorescent signal after photo-cleavage. The third moiety is an anticancer prodrug, SN-38, which also acts as a fluorescent group for imaging (monitoring). SN-38 is an active metabolite of irinotecan, a topoisomerase I inhibitor that inhibits DNA unwinding^23^. Upon light activation in a cancer cell, **PT-1** interferes with DNA religation, thus causing DNA damage that leads to apoptosis^24^. *In vitro* and *in vivo* studies reveal significant inhibition of cancer growth by **PT-1** upon irradiation with 405 nm laser light.

## Results and Discussion

### Synthesis, fluorescence changes, and the mechanism of activation of PT-1

**Figure 1:**
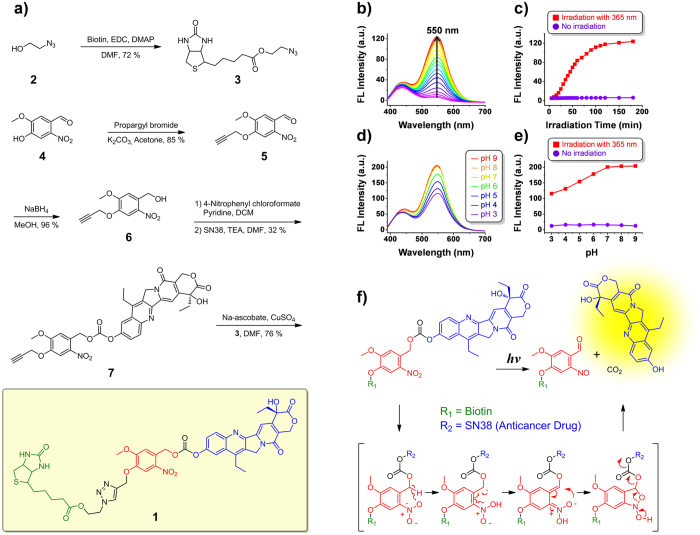
Synthesis, fluorescence changes, and the proposed mechanism of activation of PT-1. (**a**) The synthesis of **PT-1**. (**b**) Fluorescence spectra of a 10 μM solution of **PT-1** in PBS (pH 7.4) during a 3 h period in the presence of 365-nm ultraviolet (UV) light. A.U.: arbitrary units. (**c**) Time course of changes in fluorescence intensity at 365 nm. (**d**) Fluorescence spectra of a 10 μM solution of **PT-1** in PBS at various pH levels after irradiation for 3 h. (**e**) Comparison of irradiated and non-irradiated samples. (**f**) Proposed drug release mechanism of **PT-1** upon 365 nm photo-irradiation.

The synthesis of **PT-1** is shown in Fig. 1a. 2-Azidoethanol **2** and compound **4** were synthesized by previously reported methods^25^,^26^. Compound **2** was coupled with biotin in the presence of EDCI to produce compound **3** in moderate yield. Reaction of compound **4** with propargyl bromide yielded alkyne **5**, which on reduction with sodium borohydride gave the functionalized benzyl alcohol **6**. The latter compound was then treated with 4-nitrophenyl chloroformate with SN-38 to give intermediate **7**. A “click” reaction between compounds **7** and **3** then yielded the desired target **PT-1**. All new compounds (**PT-1** and 2–7) were characterized by ^1^H and ^13^C nuclear magnetic resonance (NMR) spectroscopy and electrospray ionization mass spectrometry (Fig. S6–19 in Supporting Information).

**PT-1** incorporates an *o*-nitrobenzyl (nitrovanillin) subunit that was designed to act as a light-triggered unit to permit photo-activation in cancer cells. Photocaged molecules derived from o-nitrobenzyl are especially valuable for cellular studies because they and their daughter species typically induce negligible damage to biomolecules upon irradiation with 365-nm ultraviolet light. Therefore, to activate **PT-1** it was irradiated at 365 nm using a commercially available UV hand lamp. **PT-1** is stable on the laboratory time scale and, in the absence of UV irradiation, is characterized by a weak fluorescence emission whose maximum falls at 550 nm (Fig. 1c). Upon irradiation at 365 nm, a strong fluorescence emission feature at 550 nm is observed (Fig. 1b,c). The increase in fluorescence emission intensity is consistent with photoinduced cleavage of the fluorescent SN-38 subunit from the **PT-1** core under the influence of light. The photo-activation of **PT-1** was explored as a function of time. On the basis of these experiments we conclude that essentially complete drug release occurs within 2 h after the irradiation process is initiated (Fig. 1c). The proposed mechanism of activation and release of SN-38^27^ is shown in Fig. 1f.

Intracellular thiols are present in excess in cancer cells. Their ability to interfere with the photo-release of SN-38 was evaluated by subjecting **PT-1** to irradiation at 365 nm in the presence of various thiols (cysteine, homocysteine, or glutathione). The same changes in fluorescence were observed as those in their absence (Fig. S3 in Supporting Information), leading us to discount thiol-based interference as a cause for concern.

Given the disparities in pH between cancer cells and most normal healthy cells, we evaluated whether the fluorescent behavior of **PT-1** (10 μM aqueous) was pH dependent. As shown in Fig. 1d,e, there was no appreciable change in the fluorescence intensity of **PT-1** over the 3 to 9 pH range in the absence of irradiation at 365 nm. This finding leads us to suggest that **PT-1** would display high stability over the full biologically relevant pH range when applied *in vitro* or *in vivo*. After irradiation, however, the fluorescence intensity of **PT-1** increased over the 3 to 9 pH range, with the greatest increase being seen once a pH of ca. 7 was reached. We conclude that **PT-1** undergoes photo-activation over a wide pH range. The activation of **PT-1** and release of SN-38 was analyzed by high performance liquid chromatography (HPLC) and mass spectrometry (MS). The HPLC chromatogram showed one peak corresponding to **PT-1** eluting at 27 min in the absence of irradiation. A new peak corresponding to SN-38 appeared at 17.5 min after UV irradiation (Fig. S4 in Supporting Information). These findings served to confirm that photo-irradiation of **PT-1** leads to the clean production of SN-38 (Fig. S5 in Supporting Information).

### *In vitro* bioimaging, cytotoxicity, and the mode of action of PT-1

**Figure 2:**
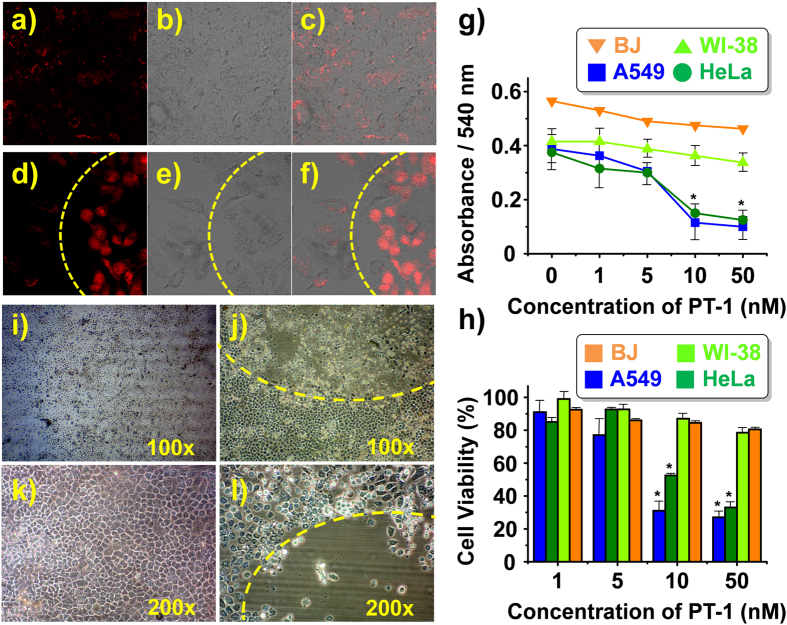
*In vitro* bioimaging and cytotoxicity of PT-1. (**a–f**) Intracellular fluorescence intensity recorded for A549 cells 1 h after incubation with of **PT-1** in the absence (**a–c**) of and (**d–f**) after irradiation with 405 nm light for 1 h over the area delineated by the yellow dashed line. (**g**) Cytotoxicity of **PT-1** observed in two cancer cell lines (A549 and HeLa) and two human normal fibroblast cell lines (WI-38 and BJ) after photo-activation. (**h**) Cell viability (relative percentage) observed for the A549, HeLa, WI-38, and BJ cell lines after treatment with **PT-1** (incubation for 1 h) followed by irradiation at 405 nm for 1 h. Viability was determined via a 3-(4,5-dimethythiazol-2-yl)-2,5-diphenyl tetrazolium bromide (MTT) assay performed 24 h after photo-irradiation. *P < 0.05. (**i–l**) Phase contrast images of A549 cancer cells undergoing apoptosis. In these studies, the cells were incubated with 10 nM **PT-1** for 30 min followed by irradiation with 405 nm laser light for 1 h over the area indicated by the yellow dashed lines (**j**,**l**).

Biotin is essential for normal cellular growth, function, and proliferation. Various cancer cell lines, including breast cancer cells (MDA-M231, MCF7), human lung cancer cells (A549), cervical cancer cells (HeLa), hepatic cancer cells (HepG2, Huh7, Hep3B), stomach cancer cells (NCI-N87, AGS), prostate cancer cells (Du145, PC3), pancreatic cancer cells (Panc-1), leukemic cells (L1210FR), mastocytoma (P815), lung (M109), renal (RENCA, RD0995), colon (Colo-26), and ovarian cancer cells (Ov2008, ID8), express greater levels of biotin receptors than do healthy cells^28^; therefore, biotin uptake is much higher in cancer cells. Accordingly, we utilized biotin as a cancer cell-targeting agent in the design of **PT-1**. For *in vitro* analysis of **PT-1** in the presence and absence of irradiation, we utilized 12 biotin receptor-positive cancer cell lines (A549, HeLa, MDA-M231, A549, Huh7, Hep3B, Du145, NCI-N87, AGS, HepG2, MCF7, PC3, and Panc-1) and 3 biotin receptor negative normal cell lines (BJ, WI-38 and 293T, Fig. S21 and Table S1 in Supporting Information). To test the localization of **PT-1** *in vitro*, biotin receptor-positive cells (A549 and HeLa) and biotin receptor negative cells (WI-38 and BJ cells) were studied in detail. The A549 and HeLa cells were incubated with **PT-1** (10 nM) for 1 h and then irradiated with a 405-nm laser. In the absence of irradiation, no significant fluorescence was observed (Fig. 2a–c). However, the emission intensity increased considerably upon irradiation (Fig. 2d–f). Cell viability assays were also performed to evaluate the cytotoxicity of **PT-1** and two reference compounds (ref. 7: without a biotin unit, and ref. 8: without a photo-releasing unit) toward various cancer and healthy cells (Figs S21–23 and Table S1 in Supporting Information)^28^. Statistically significant higher cytotoxicity was observed for the cancer cells than in the case of healthy cells upon incubation with 10 nM **PT-1** followed by irradiation at 405 nm (Figs 2g,h, and S21). Viabilities for both cancer and healthy cells were similar for refs 7 and 8, a finding that is consistent with the cancer targeting effect of biotin (see Fig. S23 in Supporting Information).

The phototherapeutic potential of **PT-1** was assessed by examining the population of A549 cancer cells before and after photo-irradiation. Large numbers of apoptotic cancer cells were found in the irradiated areas after 1 h but not in the untreated areas (Fig. 2j,l). Additionally, 24 h live imaging of the A459 cells was performed in the presence (Movie 2) and absence of irradiation (Movie 1). In the apoptosis-positive area no movement of A459 cells is seen, as would be expected for an apoptosis process that occurs after unmasking of the active SN-38 subunit from the **PT-1** core. Cancer cells originally present in the non-irradiated area (left side) showed the hallmarks of efficient survival. However, those that migrated into the irradiated area were found to undergo apoptosis (Movie 2). On this basis, we believe that **PT-1** is selectively taken up by cancer cells *via* biotin targeting and induces apoptosis upon photo-irradiation.

**Figure 3:**
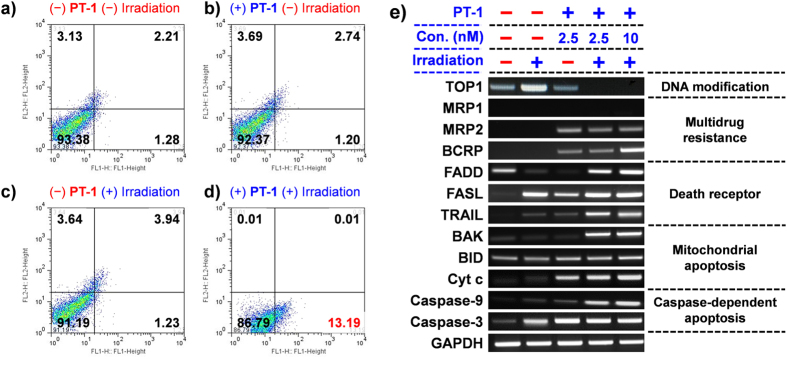
Fluorescence-activated cell sorting (FACS) analysis and evidence for the proposed mechanism of PT-1 action. (**a–d**) Efficacy of **PT-1** against A549 cells observed in the presence and absence of irradiation using a 405-nm laser for 1 h, as inferred from propidium iodide (PI) staining 12 h after irradiation. (**e**) Expression of topoisomerase I, various drug transporters, mitochondrial apoptotic genes, death cell receptors, and caspase apoptotic genes according to reverse-transcription polymerase chain reaction analysis of A549 cells incubated with 0, 2.5, or 10 nM **PT-1** for 30 min, followed by irradiation with 405-nm light for 1 h. Cells treated 10 nM SN-38 were used as controls. Cell lysates were collected 24 h after the laser irradiation.

Further analyses were carried out using fluorescence-activated cell sorting (FACS) in conjunction with PI (propidium iodide) staining (Fig. 3a–d). The number of apoptotic cells induced by SN-38 (12.5 nM) was found to be 10%^29^. In the absence of irradiation, the number of apoptotic cells induced by **PT-1** (10 nM) was 1.20% (Fig. 3b). This number increased to 13.19% upon irradiation of the **PT-1** (10 nM) cells for 1 h (Fig. 3d). Thus, **PT-1** becomes a stronger chemotherapeutic than SN-38 upon irradiation. This is as expected for a system with cancer-localizing capability. In agreement with the FACS results, the fluorescent and phase contrast images of cells revealed evidence of effective apoptosis in the case of the A459 cells induced by **PT-1** after 24 h (Fig. S24 in Supporting Information).

In general, SN-38, an active metabolite of irinotecan, inhibits DNA topoisomerase I by intercalating into the DNA strand and disrupting DNA replication. To validate and compare the mode of action of **PT-1** and SN-38, we examined the expression of topoisomerase I and various genes, including mitochondrial apoptotic genes, genes associated with cell death receptors, caspase enzymes, and a multidrug resistance (MDR) protein involved in the apoptosis of cancer cells (Fig. 3e). A459 cells showed diminished expression of topoisomerase I after treatment with **PT-1** followed by irradiation (Fig. 3e). In contrast, no significant change in the expression of topoisomerase I was observed without irradiation or in the absence of **PT-1**. Taken in concert, these findings are consistent with the suggestion that upon irradiation **PT-1** produces a species that has a mode of action similar to that of independently administered SN-38. Given the HPLC and MS analyses discussed above, it is a reasonable proposition that it is SN-38 itself that is released following photo-irradiation *in vitro*.

Cell death receptors, such as FADD, FASL, and TRAIL, are surface-bound receptors that contribute significantly to the induction of apoptosis by specific ligands^30^. The activation of death receptors quickly activates the caspase-dependent apoptotic genes; therefore, this apoptotic pathway is rapid. Cell death receptors (FADD, FASL, and TRAIL) were upregulated upon treatment with **PT-1** followed by irradiation (Fig. 3e). In contrast, in the absence of irradiation or added **PT-1**, insignificant changes in expression were observed. We thus conclude that the combination of **PT-1** and irradiation serves to modulate the TRAIL receptor in cancer cells and enhance their susceptibility to apoptosis.

Along with surface death receptors, we observed an increase in mitochondria related apoptotic genes, such as BAK, BID, and CytC, after **PT-1** treatment and irradiation (Fig. 3e); this upregulation stimulates mitochondrial dysfunction and eventually leads to apoptosis. Furthermore, the mitochondrial intermembrane protein cytochrome c showed enhanced expression after light activation in the presence of **PT-1**. Cytochrome c is known to stimulate apoptosis *via* activation of caspase 9 and 3 (Fig. 3e)^31^. Higher levels of caspases 3 and 9 were seen after treatment with **PT-1** and irradiation. The upregulation of the relevant mitochondrial proteins, cell surface receptors, and caspases is taken as evidence that **PT-1** is able to induce apoptosis efficiently in cancer cells following irradiation.

A major challenge associated with chemotherapy is the induction of multidrug resistance. Various multidrug resistance proteins are up- regulated upon treatment with antitumor drugs. Therefore, we evaluated the effect of light-activated **PT-1** on the multidrug resistance protein (MRP2) and breast cancer resistance protein (BCRP). Even though their expression increased in the presence of **PT-1** and light, an enhanced antitumor effect was seen in the case of A459 cells. Presumably, this desired effect reflects the benefit of the biotin-based targeting.

### *In vivo*/*ex vivo* chemotherapeutic effect and diagnosis of PT-1 in xenograft mouse models

**Figure 4:**
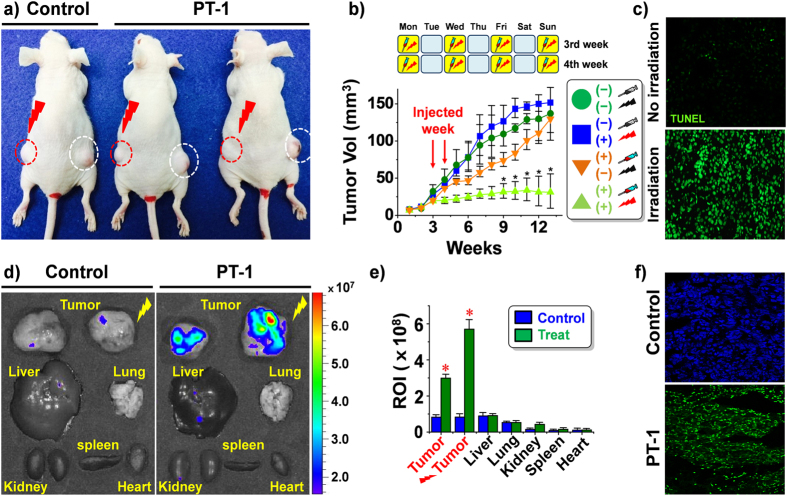
Chemotherapeutic effect and imaging utility of PT-1 as seen in *in vivo*/*ex vivo* xenograft mouse models. Three weeks after inoculation of mice with the A549 human lung carcinoma cells, the resulting xenograft mice were injected with PBS or **PT-1** (8 mg/kg body weight, eight doses) *via* the tail vein every other day for two weeks. *In vivo* images designed to characterize the chemotherapeutic effects were recorded 12 weeks after the initial inoculation. (**a**) Representative *in vivo* images of xenograft mice injected with **PT-1** and non-injected control. After the tail vein injection, the tumors on the left-side of the animals were irradiated with a 405-nm laser for 1 h. The right-side tumors (without exposure to light) served as controls (n = 8 per each treatment). (**b**) Quantitative analysis of tumor volume with/without irradiation with 405-nm laser light. (**c**) Fluorescent images of TUNEL assay (green). Cryosectioned tumor tissues without irradiation (upper panel) and irradiated tumor tissue (lower panel). (**d**) Fluorescent *ex vivo* images of tumors and various organs of non-injected control (left side) and **PT-1**-injected xenograft mice (right side). The images were taken 3 h after tail vein injection and irradiation with 405-nm laser light for 1 h. (**e**) Quantitative analysis of the fluorescence intensity of regions of interest (ROI) of tumors and various dissected organs. (**f**) Fluorescent images of cryosectioned tumor tissues (upper panel, blue: 4′,6-diamidino-2-phenylindole (DAPI)-stained control tumor tissue and lower panel, green: fluorescent images of **PT-1**). Magnification for c and f: 200x *P < 0.05.

The strong antitumor effect of **PT-1** observed in the above *in vitro* experiments prompted us to examine its *in vivo* chemotherapeutic potential in tumor models. This was done by monitoring tumor growth in A549 cell-inoculated (xenograft) mice bearing tumors on both flanks. **PT-1** was administered by tail vein injection (eight doses). The left-side tumor was subject to irradiation using a 405 nm laser (1.4 W/cm^3^) 3 h after the tail vein injection, while the right-side tumor was not irradiated (Fig. 4a). Under conditions of photo-irradiation **PT-1** suppressed tumor growth. This is reflected in the volume of the left-side tumor being significantly smaller than the right-side tumor (which was not subject to irradiation) or in the control animals (which did not received **PT-1** but were still subject to irradiation; cf. Fig. 4b and S25 for comparison between control, SN-38, and **PT-1**; 3 mg/kg of each compound were treated to mice). These results are consistent with photo-activation of **PT-1** occurring within the cancer cells and the concomitant release of SN-38, which then leads to apoptosis as by confirmed TUNEL assay and PI-staining (Figs 4c and S26). A strong fluorescent signal derived from **PT-1** was observed in the photo-irradiated tumor tissues (Fig. 4d,e). The distribution of **PT-1** in various organs and its tumor-targeting capacity were evaluated by subjecting dissected organs and tumor tissue to *ex vivo* imaging; this was done with control (untreated) and **PT-1** treated mice (Fig. 4c,d). A strong fluorescent signal was detected only in the tumor sections of the treated mice; no other organs, including the kidneys, lungs, spleen, liver, and heart, produced an appreciable fluorescence signal (Fig. 4c,d). The small increase in fluorescence in the liver independent of irradiation is due to residual fluorescence from endogenous pigments present in the gallbladder which overlay with a portion of the liver image. In our studies, the gallbladder was not removed from the liver tissues; therefore, an inherent (endogenous) fluorescence signal is observed. Cryosectioned tumor tissues subject to irradiation following tail vein injection of **PT-1** were also characterized by a stronger fluorescence signal than seen in the untreated, control tumor tissues (Fig. 4f). On this basis we conclude that **PT-1** 1) achieves good tumor targeting, 2) is selectively activated by light, and 3) releases SN-38 into the tumor tissues. It is also highly effective at preventing tumor regrowth under conditions of photo-irradiation.

**Figure 5:**
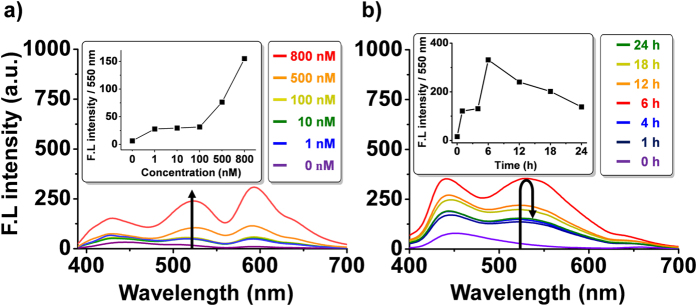
Fluorescence-based studies of the *in vitro* and *in vivo* dynamic behaviour of PT-1. (**a**) Concentration-dependent change in the fluorescent intensity of intracellular **PT-1**. In this study, A549 cells were treated with various concentration of **PT-1** for 1 h and the cells were washed with PBS. Fresh culture media was added and the cells were irradiated for 1 h. After 24 h, the cells were washed with PBS and dissolved in DMSO. The fluorescent intensity was then measured. (**b**) Time course studies of the fluorescent intensity of **PT-1** in sera obtained from xenograft mice. After tail vein injecting with **PT-1** (8 mg/kg, single dose), sera aliquots were collected at various times (0, 1, 4, 6, 12, 18, and 24 h) and then irradiated with 405 nm laser for 1 h before the fluorescent intensities at 550 nm, were measured (n = 4 per time-point); λ_ex_ 365 nm; slit 10/10.

Insights into the pharmacokinetic features of **PT-1** were obtained by monitoring changes in its fluorescent intensity as a function of time and dose, both *in vitro* and *in vivo*. In one experiment, A549 cells were treated with various concentration of **PT-1** for 1 h. The cells were then irradiated for 1 h and the fluorescent intensity measured after 24 h. The intensity was found to depend upon the dose of **PT-1** (Fig. 5a). Separately, xenograft mice were tail vein injected with **PT-1** (8 mg/kg, single dose) and serum samples collected at various time intervals (0, 1, 4, 6, 12, 18, and 24 h). These were then irradiated with a 405 nm laser for 1 h and the fluorescent intensity recorded (n = 4/ time-point). In the case **PT-1**, the greatest fluorescent intensity was seen at 6 h (Fig. 5b) and it was very stable in serum (Fig. S27 in Supporting Information). This is consistent with this probe having a longer circulation *in vivo* than SN-38, which has a half-life (t_1/2_) of 6.38 h in mouse plasma^32^. Thus, at 6 h, where the serum concentration of **PT-1** is maximal, significant quantities of SN38 have already been cleared from the animals. We believe that this differential response reflects the presence of the targeting unit **PT-1**, which serves not only to increase cancer targeting but also to enhance the plasma circulation lifetime relative to SN-38.

## Conclusions

The present study has served to demonstrate that it is possible to design systems that target tumors, which are readily activated by application of an external photo-stimulus, and which allow monitoring via optical means. The combination of photo-activation and biotin-based targeting provides a high level of specificity for cancer cells relative to healthy cells *in vitro*, an effect that is mirrored *in vivo* in the case of A549 mouse xenografts. We thus propose that the present phototheranostic strategy can be extended beyond the specific components present in **PT-1** to provide a new, generalizable approach to the development of rationally designed small molecular cancer chemotherapeutics.

## Methods

### Cell culture and probe treatment

Twelve biotin receptor-positive cell lines; human lung carcinoma cells (A549), human cervical cancer cells (HeLa), human breast cancer cells (MCF7, MDA-M231), human liver cancer cells (HepG2, Huh7, Hep3B), human prostate cancer cells (Du145, PC3), human gastric cancer cells (NCI-N87, AGS), human pancreatic cancer cell (Panc-1), and a biotin receptor-negative cell: human normal embryonal kidney epithelial cell (293T) were purchased from the Korean Cell Line Bank (Seoul, Republic of Korea). Two more biotin receptor-negative normal cell lines, human normal fibroblast cells obtained from fetal lung (WI-38 cells) or neonatal foreskin (BJ) cells were purchased from the Korean Cell Line Bank (Seoul, Republic of Korea) or Modern Cell & Tissue Technologies (MCTT, Seoul, Republic of Korea). The cells were cultured in either Roswell Park Memorial Institute medium (RPMI-1640, GIBCO BRL, Grand Island, NY, USA) or Dulbecco’s Modified Eagle’s Medium (DMEM, GIBCO BRL) supplemented with 10% fetal bovine serum (FBS, GIBCO), and 1% penicillin and streptomycin (GIBCO), at 37 °C in a humidified atmosphere containing 5% of CO_2_. When the cell density reached 70–80% of confluence, subculturing was considered complete. The medium was changed approximately every 3 to 4 days.

### Cytotoxicity analysis

Three normal cells lines (WI-38, BJ, and 293T) and twelve biotin receptor-positive cell lines were used to evaluate the cytotoxicity of **PT-1**. Prior to test, the cells were washed two times with PBS and then exchanged into FBS-free culture medium. Actual cell viability was monitored by using a 3-(4,5-dimethylthiazol-2-yl)-2,5-diphenyltetrazolium bromide (MTT; Life Technologies, Carlsbad, CA, USA) assay in accord with the manufacturer’s instructions. Briefly, 1.5 × 10^4^ cells were seeded in each well in a 24-well plate. The next day, the culture medium was removed and exchanged with a fresh medium (1 ml) containing different concentrations (0, 10, 100, 800, or 1000 nM) of **PT-1**. Cells were incubated at 37 °C for 24 h. Then, 12 mM MTT solution (600 μl) was added to each well. For negative controls 500 μl of the MTT stock solution and distilled water were added per well in the absence of **PT-1**, i.e., to 500 μl of the medium alone. The medium of each well was removed after 24-h incubation, and 300 μl of DMSO was added. The resulting suspension was mixed thoroughly for 15 min and the absorbance was monitored using a microplate spectrophotometer at 540 nm (PowerWave XS, Bio-Tek, Winooski, VT, USA). The cytotoxicity of **PT-1** in A549 and HeLa cells was confirmed to be >100 μM without photo-activation. Cell treated with 0, 1, 5, 10, or 50 nM **PT-1** for 1 h were then subject to an hour of 405-nm irradiation. MTT assays were carried out as above.

### *In vitro* cell imaging

Prior to fluorescent imaging, cells were seeded in 35-mm confocal dishes (glass bottom dish, SPL, Kyong-Gi, Republic of Korea) and allowed to stabilize for 48 h. The cell density was 2.0 × 10^6^ in the 35-mm confocal dish. The A549 and HeLa cells were treated with **PT-1**. The cells were incubated with media containing 10 nM **PT-1** (in 10 μl of DMSO in 2 of mL media per 35-mm dish) for 1 h at 37 °C in 5% CO_2_. Then, 1 ml of PBS was added twice to wash the cells, prior to adding FBS-free DMEM culture medium. The cells were again washed twice with PBS, and florescent images were taken under a confocal laser scanning microscope (Carl-Zeiss LSM 5 Exciter, Oberko, Germany), which was equipped with a 405-nm Argon laser and 500-nm pass filter. Thus the effects of photo-irradiation or its absence could be evaluated.

Analysis of PI-positive apoptotic cells was performed according to the manufacturer’s instructions. PI is a late apoptotic dye that binds to double-stranded DNA by intercalating between base pairs. It is excited at 488 nm and emits light at a maximum of 617 nm. Briefly, A549 cells (approximately 80% confluence) were incubated with or without 10 nM **PT-1** for 30 min and then irradiated with a 405-nm laser for 1 h. The cells were incubated for an additional 24 h, before PI was added (1 μg/ml, Sigma-Aldrich) and the cells incubated for an additional 90 min at 37 °C. Stained cells were visualized by means of a Zeiss LSM510 laser scanning confocal microscope using the cyc3.5 filter (Carl Zeiss, Oberkochen, Germany).

### RT-PCR

Total RNA from the cells was isolated using the TRIzol Reagent (Invitrogen). The RNA was reverse-transcribed using a reverse transcription system (Promega, Madison, WI). EXTaq polymerase (Takara, Japan) was used for PCR amplification of different genes, under the following cycling conditions: 94 °C for 5 min; then 35 cycles of 94 °C for 30 s, 50–57 °C for 30 s, and 72 °C for 30 s; with final incubation at 72 °C for 10 min. The primers and PCR conditions are shown in Table S2.

### FACS analysis

Analysis of the PI-positive apoptotic cells was performed by flow cytometry. A549 cells were treated with 10 nM **PT-1** for 30 min with or without irradiation with the 405 nm laser for 1 h. The cells were further incubated for 12 h and harvested. Then the cells were washed two times with ice-cold PBS. The cells were placed (at up to 10^6^/ml) in BD FACS tubes and incubated with 5 mg/ml PI (Sigma-Aldrich) in PBS with 10% FBS for 30 min at room temperature. The cells were then washed in ice-cold PBS. The PI fluorescence was measured in the FL-1 channel of a FACSAria instrument (BD, San Jose, California, USA).

### Mouse xenograft model

To examine chemotherapeutic effects and to obtain images of **PT-1** *in vivo*, 7- to 8-week-old BALB/c nude mice from RaonBio (Kayonggido, Yonginsi, South Korea were used as follows: control xenograft: n = 8; treatment and xenograft: n = 8). Before the various the animals were stored in an animal facility were allowed to acclimate for 48 h and maintained in accordance with the guidelines of the Care and Use of Laboratory Animals published by the National Institutes of Health (Bethesda, MD, USA). Furthermore, all animal studies were reviewed and approved by Institutional Animal Care and Use Committee (IACUC; No. 1040548-KU-IRB-16-2-A-1 and 1040548-KU-IRB-16-1-A-1) of the Korea University, Seoul, Korea. The procedures for all animal experiments were performed according to IACUC guidelines. The animals were housed in cages and provided with water ad libitum and sterilized food, and maintained in a 12 h light/dark cycle with 30–40% humidity at room temperature (21 ± 2 °C). The 354234-matrigel (BD, San Jose, California, USA) was mixed with approximately 5.0 × 10^6^ A549 cells and subcutaneously injected into the right and left flanks of the mice.

### *Ex vivo* fluorescent imaging and analysis of drug distribution in organs

The tumor targeting ability and organ distribution of **PT-1** were evaluated on the basis of *ex vivo* experiments involving the A549 cell-inoculated mice described above. To validate the theranostic effects, three weeks after the inoculation, **PT-1** in 0.1 ml of PBS was injected into the tail vein of the xenograft mice every other day for two weeks [It should be noted that (a) for theranostic effect of **PT-1** shown in Fig. 4, 8 mg/kg body weight, eight doses was used; (b) for *in vivo* kinetics of **PT-1** shown in Fig. 5, 8 mg/kg body weight, single dose was used; and (c) for comparison of therapeutic effects of **PT-1** and SN38 shown in Fig. S24, 3 mg/kg body weight was used]. **PT-1** was activated with a 405 nm laser (1.4 W/cm^3^) 3 h after the tail vein injection. *Ex vivo* spectral-fluorescence images were obtained, 2 h after the last **PT-1** injection prior to the 1-h irradiation, by means of an IVIS Lumina Series III preclinical imaging system (PerkinElmer Co., Waltham, MA, USA). The tumor tissues and various organs (lungs, heart, liver, kidneys, and spleen) of the control and injected mice were dissected to obtain *ex vivo* images. The filters used to obtain the *ex vivo* images were set to 420 and 580 nm for excitation and emission, respectively. The auto-fluorescence and fluorescent images were then deconvoluted using the manufacturer’s multiexcitation spectral analysis function. After *ex vivo* images were taken, the grafted tumor tissues were used to obtain cryosections in Tissue-Tek 100% Optimal Cutting Temperature Compound (O.C.T., Sakura Finetek, USA). The fresh tissue samples were rapidly snap-frozen in liquid nitrogen (LN_2_) or dry ice and then stored at −80 °C until used for further analysis. Freshly cryopreserved tissues were cut (6 μm slices) on an ultramicrotome at −25 °C (Leica CM 3050 S, Wetzlar, Germany). Each slice was placed on an adhesion microscope glass slide (Paul Mariendeld GmbH & Co. KG, Lauda-Königshofen, Germany). The O.C.T. compound was then removed by washing with PBS twice. The slices were incubated with 2 mM **PT-1** for 2 h with 4′,6-diamidino-2-phenylindole (DAPI) counterstaining and covered with a fluorescent slide cover glass. The fluorescent images were taken under a confocal laser scanning microscope (Carl-Zeiss LSM 5 Exciter, Oberko, Germany).

### *In vitro* and *in vivo* kinetics

A549 cells were grown up to 80% confluence in a 60 mm culture dish. The A549 cells were treated with various concentration of **PT-1** (0, 1, 10, 100, 500, and 800 nM) for 1 h and the cells were washed with PBS three times to remove any residual **PT-1**. Fresh culture media was added and the cells were photo-irradiated for 1 h. After 24 h, the cells were washed with PBS and dissolved in DMSO. The fluorescent intensity was measured. To examine *in vivo* kinetics, the fluorescent intensity of **PT-1** in mouse sera was detected. Xenograft mice were subject to a single tail vein injection with **PT-1** (8 mg/kg body weight in 0.1 ml PBS). Serum samples were collected at various time-points (0, 1, 4, 6, 12, 18, and 24 h). DMSO dissolved cell extracts and mouse serum samples were irradiated with 365 nm laser for 1 h to measure the fluorescent intensity (n = 4/time-point). Samples for absorption and emission measurements were contained in 100 μl quartz cuvettes (Sigma). Fluorescent spectra were recorded using an RF-530 PC spectrofluorometer (Shimadzu, Seisakusho, Japan) equipped with a xenon lamp. Quantification was done at 550 nm; λ_ex_ = 365 nm; slit 10/10.

### TUNEL assay

Cryosectioned tumor tissues from 8 mice were dissected and frozen sectioned (5 μm). Freshly sectioned tumor tissues were fixed with cold ethanol for 3 min and washed with PBS. TUNEL staining was performed according to the manufacture’s instruction (*In situ* cell Death Detection Kit, POD; Roche). 4′,6-diamidino-2-phenylindole (DAPI) was used for counter staining. Stained tumor tissues were imaged by means of a fluorescence microscopy (Carl Zeiss) and quantified. TUNEL-positive cells (green florescent dots) were counted at 7 randomly selected areas in the case of either irradiated or non-irradiated slides.

### Statistical analysis

The mean of each groups and standard error of the mean (SEM) were calculated from the three independent experiments carried out in triplicate. ANOVA (One-way analysis of variance) in the SAS software (version 8.2, Cary) was performed to evaluate the statistical significance differences between groups. Paired Student’s t tests were performed to compare the means when ANOVA indicated a significant difference. P values < 0.05 were assumed to denote statistical significance.

## Experimental Section

### Synthesis of compound 2

This compound was synthesized in 90% yield, according to a previously reported procedure^33^.

### Synthesis of compound 3

Biotin (100.0 mg, 0.40 mmol) was dissolved in 10 ml of DMF, and a solution of azidethanol (**2**; 71.3 mg, 0.81 mmol) in DMF (3 ml) was added to this initial solution, followed by EDC·HCl (338.2 mg, 1.63 mmol) and DMAP (75.0 mg, 0.61 mmol). The reaction mixture was stirred for 24 h at room temperature. The volatiles were removed under reduced pressure and the residue dissolved in 30 ml of CH_2_Cl_2_. The organic layer was washed with water (3 × 10 ml), concentrated *in vacuo*, and then purified by column chromatography over silica gel (MeOH/CH_2_Cl_2_:10/90) to obtain the product (92 mg) in 72% yield. ^1^H NMR (CDCl_3_, 300 MHz): δ 1.37–1.45 (m, 2H), 1.59–1.69 (m, 4H), 2.34 (t, *J* = 7.44 Hz, 2H), 267–2.71 (m, 2H), 2.82–2.88 (m, 1H), 3.07–3.13 (m, 1H), 3.44 (t, *J* = 5.22 Hz, 2H), 4.19 (t, *J* = 5.01 Hz, 2H), 4.23–4.27 (m, 1H), 4.43–4.47 (m, 1H), 5.98 (s, 1H), and 6.29 (s, 1H) ppm; ^13^C (CDCl_3_, 100 MHz): 24.9, 28.4, 33.9, 40.8, 50.0, 55.6, 60.3, 62.1, 63.1, 163.8, and 173.6 ppm; ESI-MS: the calculated value (calcd) for C_12_H_19_N_5_O_3_S (M+Na^+^): 336.11, found 336.00.

### Synthesis of compounds 4 and 5

Compound **4** was synthesized according to previously reported procedures^25^,^26^, and obtained in 60% yield. Compound 5 was synthesized using a different procedure than prescribed in the literature^34^.

### Synthesis of compound 5

Propargyl bromide (120.7 mg, 1.01 mmol) was added to a suspension of nitrovanillin (100.0 mg, 0.50 mmol) and potassium carbonate (140.3 mg, 1.01 mmol) in MeCN (20 mL). After being stirred for 6 h under reflux, the reaction mixture was concentrated under reduced pressure and diluted with 30 ml of CH_2_Cl_2_. The organic layer was washed with water (3 × 10 ml), concentrated *in vacuo*, and then purified by column chromatography over silica gel (EtOAc/hexanes: 10/90) to obtain the product (101.1 mg) in 85% yield. ^1^H NMR (CDCl_3_, 300 MHz): δ 2.63 (t, *J* = 2.4 Hz, 1H), 4.02 (s, 3H), 4.91 (d, *J* = 2.4 Hz, 2H), 7.43 (s, 1H), 7.80 (s, 1H), and 10.46 (s, 1H) ppm.

### Synthesis of compound 6

To a solution of compound **5** (100 mg, 0.42 mmol) in MeOH (10 mL) was added an excess of NaBH_4_ (64 mg, 1.70 mmol) in small portions. After 1 h, EtOAc was added to the reaction to quench the reaction. The resulting organic mixture was washed with saturated NH_4_Cl and brine, dried over MgSO_4_, and evaporated *in vacuo*. The crude product obtained in this way was purified by column chromatography over silica gel (EtOAc/hexane: 10/40) to obtain the product (96.7 mg) in 96% yield. ^1^H NMR (CDCl_3_, 300 MHz): δ 2.58 (t, *J* = 2.37 Hz, 1H), 3.99 (s, 1H), 4.82 (d, *J* = 2.34 Hz, 2H), 4.97 (t, *J* = 0.54 Hz, 2H), 7.22 (s, 1H), and 7.87 (s, 1H) ppm; ^13^C (CDCl_3_, 100 MHz): 56.7, 57.3, 63.0, 1111, 111.5, 133.6, 139.7, 145.7, and 154.7 ppm.

### Synthesis of compound 7

To a DMF (5 ml) solution of compound **6** (50 mg, 0.21 mmol), was added 4-nitrophenyl chloroformate (46.4 mg, 0.23 mmol) and pyridine (33.3 mg, 0.42 mmol) at 0 °C. After allowing the reaction mixture to warm to room temperature, it was stirred for an additional 12 h at room temperature. At this juncture, 7-ethyl-10-hydroxy-camptothecin (51.4 mg, 0.42 mmol) and TEA (42.6 mg, 0.42 mmol) in DMF (5 mL) were added at room temperature under argon. The mixture was then stirred at room temperature for 24 h before the reaction mixture was mixed with CH_2_Cl_2_, and water. The organic layer was separated, dried over MgSO_4_, and concentrated *in vacuo*. Purification by column chromatography over silica gel (MeOH/CH_2_Cl_2_: 10/90) produced 32 mg of the desired product corresponding to a 32% yield. ^1^H NMR (DMSO-*d*_6_, 400 MHz): δ 0.84 (t, *J* = 7.16 Hz, 3H), 1.25 (t, *J* = 7.48 Hz, 3H), 1.76–1.88 (m, 2H), 3.64 (t, *J* = 2.32 Hz, 1H), 3.88 (s, 3H), 4.95 (d, *J* = 2.24 Hz, 2H), 5.28 (s, 2H), 5.40 (s, 2H), 5.61 (s, 2H), 6.52 (br s, 1H), 7.27 (s, 1H), 7.29 (s, 1H), 7.75 (dd, *J* = 9.16, 2.48 Hz, 1H), 7.84 (s, 1H), 8.13 (d, *J* = 2.52 Hz, 1H), and 8.18 (d, *J* = 9.16 Hz, 1H); ^13^C (DMSO-*d*_6_, 100 MHz): 8.4, 14.5, 21.4, 22.9, 30.9, 50.2, 57.0, 57.2, 65.9, 67.7, 73.0, 78.9, 80.0, 97.5, 111.0, 112.8, 115.7, 119.8, 125.6, 126.3, 127.7, 129.4, 132.2, 140.2, 146.2, 146.4, 146.5, 147.3, 149.9, 150.7, 152.9, 153.1, 154.2, 157.5, and 173.2 ppm.

### Synthesis of compound PT-1

Compound **3** (26.2 mg, 0.08 mmol), **7** (50 mg, 0.07 mmol), CuSO_4_·5H_2_O (0.38 g, 0.15 mmol), and sodium ascorbate (0.30 g, 0.15 mmol) were mixed in DMF (5 ml) and then stirred at room temperature under argon for 12 h. At this point, the volatiles were under reduced pressure and the reaction mixture was purified by HPLC to give the desired product (56 mg; 76% yield). ^1^H NMR (DMSO-*d*_6_, 400 MHz): δ 0.87 (t, *J* = 7.32 Hz, 3H), 1.27 (t, J= 7.6 Hz, 5H), 1.38–1.50 (m, 6H), 1.52–1.91 (m, 3H), 2.24 (t, *J* = 7.24 Hz, 2H), 2.52–2.58 (m, 4H), 2.77 (dd, *J* = 12.52, 5.04 Hz, 1.5H), 3.03–3.19 (m, 5H), 3.88 (s, 3H), 4.09–4.12 (m, 1.5H), 4.27–4.30 (m, 1.5H), 4.42 (t, *J* = 5.08 Hz, 2.5H), 4.66 (t, *J* = 4.96 Hz, 2H), 5.29 (s, 3H), 5.42 (s, 1.5H), 5.63 (s, 1.5H), 6.38 (s, 3H), 6.43 (s, H), 6.57 (s, 1.5H), 7.28 (s, 1H), 7.32 (s, 1H), 7.77 (dd, *J* = 9.12, 2.4 Hz, 1H), 7.99 (s, 1H), 8.14 (d, *J* = 2.4 Hz, 1H), 8.20 (s, 0.5H), 8.22 (s, 0.5H), and 8.3 (s, 1H) ppm; ^13^C (DMSO-*d*_6_, 100 MHz): 8.4, 14.5, 22.9, 24.9, 28.6, 30.9, 33.7, 49.4, 50.2, 55.9, 56.9, 59.9, 61.7, 62.6, 62.8, 65.9, 67.7, 73.0, 97.5, 110.6, 112.7, 115.7, 119.8, 125.5, 125.7, 126.1, 127.7, 129.3, 140.4, 142.5, 146.2, 146.4, 147.2, 147.5, 149.9, 150.7, 152.8, 153.2, 154.1, 157.5, 163.5, and 173.2; ESI-MS: calcd for C_46_H_48_N_8_O_14_S (M+H) was 969.40; detected 969.40.

### Synthesis of compound 8

EDC.HCl (79 mg, 0.51 mmol) was added to a solution of 7-ethyl-10-hydroxycomapothecin (100 mg, 0.25 mmol), biotin (74 mg, 0.30 mmol) and DMAP (62 mg, 0.51 mmol) in DMF (5 ml) at room temperature. After 8 h the mixture was washed with an aqueous 1*N* HCl solution, the organic layer was dried with MgSO_4_, filtered and concentrated *in vacuo*. Purification by column chromatography yielded 95 mg (61% yield) of the title product. ^1^H NMR (DMSO-*d*_6_, 400 MHz): δ 0.85 (t, *J* = 7.2 Hz, 3H), 1.24 (t, *J* = 7.4 Hz, 3H), 1.52 – 1.41 (m, 3H), 1.72 – 1.65 (m, 2H), 1.84 (quint., *J* = 6.92 Hz, 2H), 2.61 (d, *J* = 12.64 Hz, 1H), 2.64 (t, *J* = 7.28 Hz, 1H), 2.84 (dd, *J* = 12.44, 4.96 Hz, 2H), 3.17 – 3.08 (m, 3H), 4.37 – 4.33 (m, 2H), 5.22 (s, 2H), 5.40 (d, *J* = 9.12 Hz, 1H), 6.46 (d, *J* = 33.64 Hz, 3H), 6.64 (s, 2H), 7.33 (s, 2H), 7.60 (dd, *J* = 9.24, 2.2 Hz, 2H), 7.89 (d, *J* = 2.32 Hz, 1H), 8.15 (d, *J* = 9.12 Hz, 2H).

## Materials and Methods for Synthesis

The reagents used in this study were purchase from Alfa-Aesar, Aldrich, TCI, Carbsynth, Ducsan, and Acros without further purification. Silica gel 60 (Merck, 0.040–0.063 mm) was used for column chromatography and Merck 60 F254 silica gel plates were used for analytical thin-layer chromatography. ^1^H and ^13^C NMR spectra were recorded in CDCl_3_ or DMSO-*d*_6_ on Varian instruments.

## Additional Information

**How to cite this article**: Shin, W. S. *et al*. Programmed activation of cancer cell apoptosis: A tumor-targeted phototherapeutic topoisomerase I inhibitor. *Sci. Rep*. **6**, 29018; doi: 10.1038/srep29018 (2016).

## Supplementary Material

Supplementary Information

Supplementary Movie S1

Supplementary Movie S2
