# Supplementary material for: Programmed activation of cancer cell apoptosis: A tumor-targeted phototherapeutic topoisomerase I inhibitor
Source: Sci Rep. 2016 Jul 4;6:29018. doi: 10.1038/srep29018 (PMC4931442; doi:10.1038/srep29018)
Supplement: Supplementary Information [file srep29018-s1.pdf]

Supplementary information

Programmed activation of cancer cell apoptosis: A tumor-targeted phototherapeutic topoisomerase I inhibitor

Weon Sup Shin,[1‡] Jiyou Han,[2‡] Rajesh Kumar,[1‡] Gyung Gyu Lee,[2] Jonathan L. Sessler,[3]* Jong-Hoon Kim,[2]* and Jong Seung Kim[1]*

[1] Department of Chemistry, Korea University, Seoul 136-701, Korea

[2] Division of Biotechnology, Laboratory of Stem Cells and Tissue Regeneration, College of Life Sciences & Biotechnology, Korea University, Seoul 136-713, Republic of Korea

[3] Department of Chemistry, University of Texas at Austin, Austin, TX 78712-1224, USA

‡ Equal contribution to this work

* E-mail: sessler@cm.utexas.edu (J. L. Sessler) jhkim@korea.ac.kr (J. H. Kim), jongskim@korea.ac.kr (J. S. Kim)

Movie legends

Supplementary movie 1. Live imaging of the A459 cells (24 h) in the absence of irradiation (Movie 1).

Supplementary movie 2. Live imaging of the A459 cells (24 h) in the presence of irradiation (Movie 2).

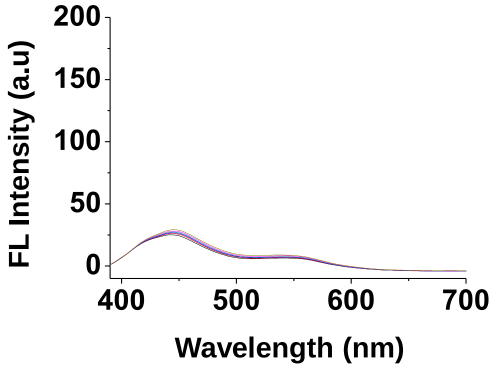

Figure S1. Fluorescence spectra of a 10 μM solution of **PT-1** recorded before and after being held in a pH 7.4 PBS buffer solution for 3 h in the absence of 365 nm UV light.

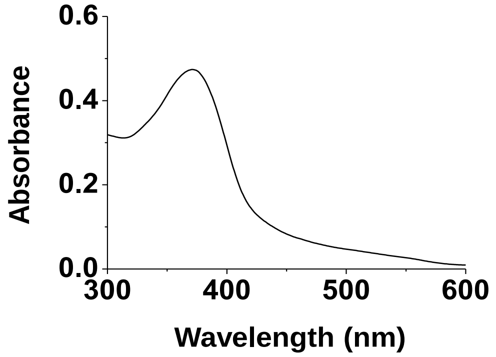

Figure S2. Absorption spectrum of **PT-1** (20 μM, 2% DMSO, and 98% PBS buffer).

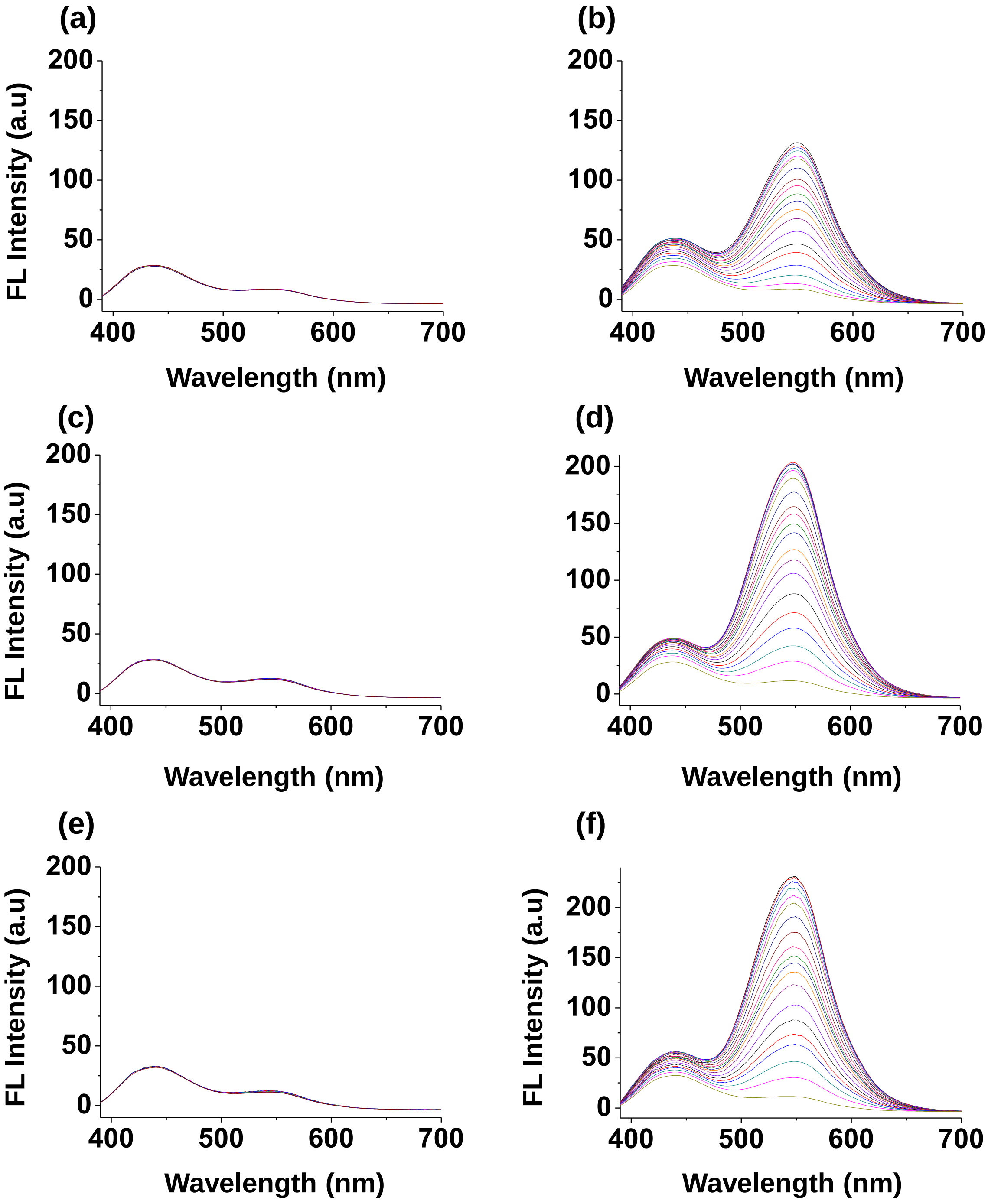

Figure S3. Fluorescence spectra of 10 μM solution of **PT-1** in a pH 7.4 PBS buffer solution recorded after 3 h and in the (a) absence of UV light with the solution containing 1 mM GSH, (b) presence of UV light with the solution containing 1 mM GSH, (c) absence of UV light with the solution containing 1 mM Cys, (d) presence of UV light with the solution containing 1 mM Cys, (e) absence of UV light with the solution containing 1 mM homocysteine (Hcy), and (f) presence of UV light with the solution containing 1 mM Hcy.

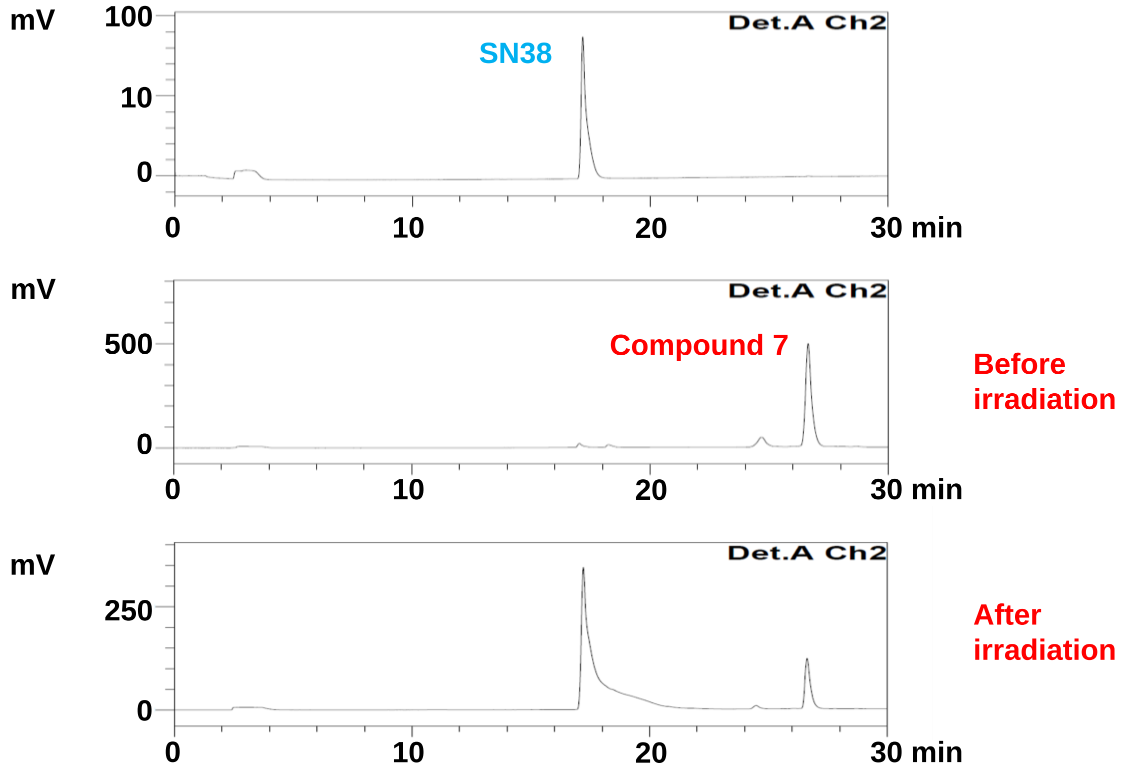

Figure S4. HPLC analysis of **PT-1** and its putative photodegradation. HPLC conditions: Wavelength: 365 nm; gradient: 20% acetonitrile to 80% acetonitrile in choloroform for 15 min, then 100% acetonitrile for 10 min.

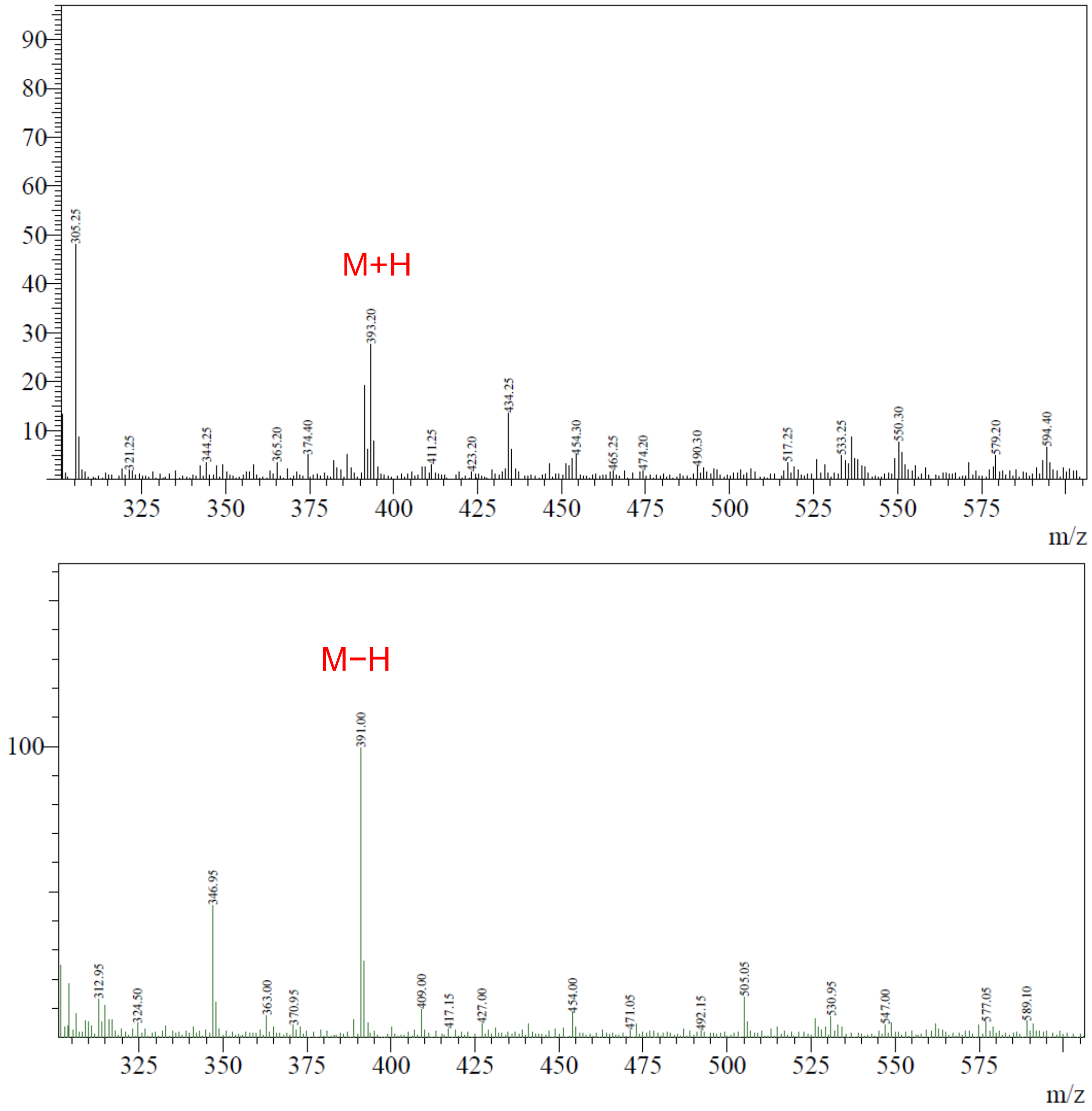

Figure S5. MS analysis of **PT-1** after irradiation at 365 nm.

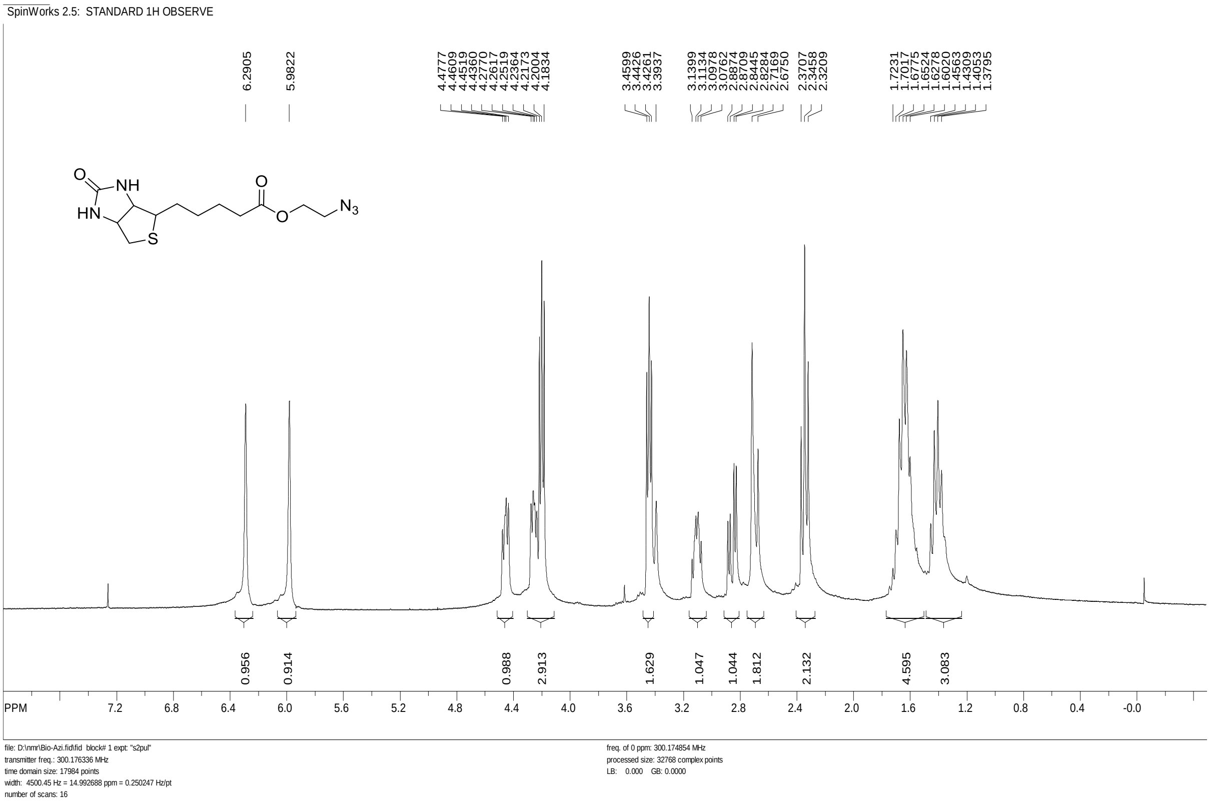

Figure S6. ^{1}H NMR spectrum of **3.**

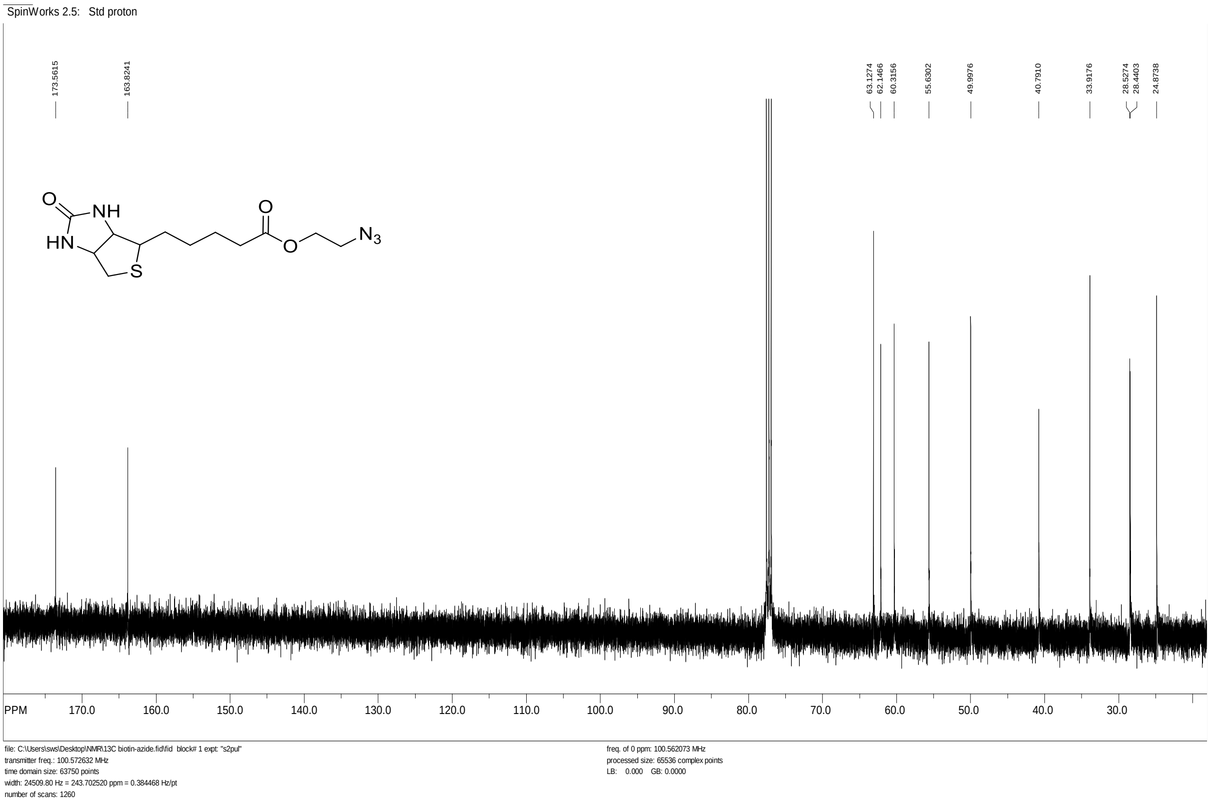

Figure S7. ^{13}C NMR spectrum of **3.**

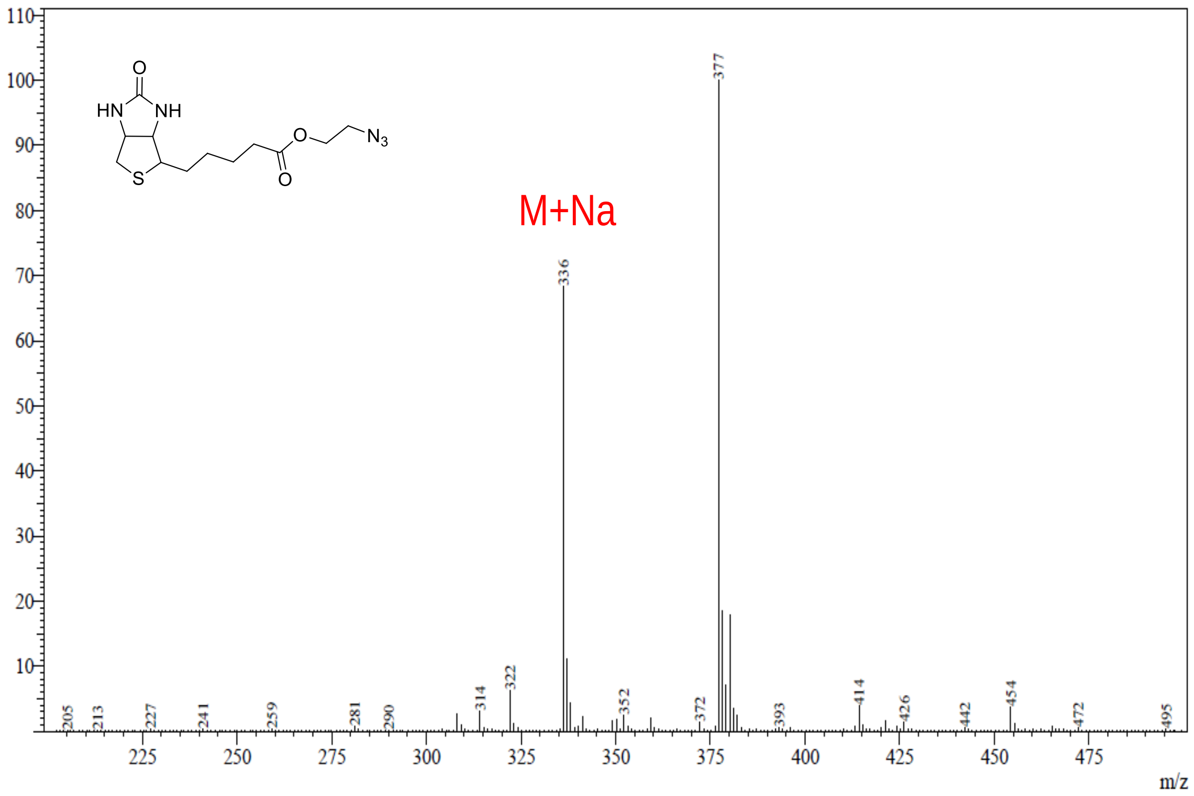

Figure S8. MS spectrum of **3.**

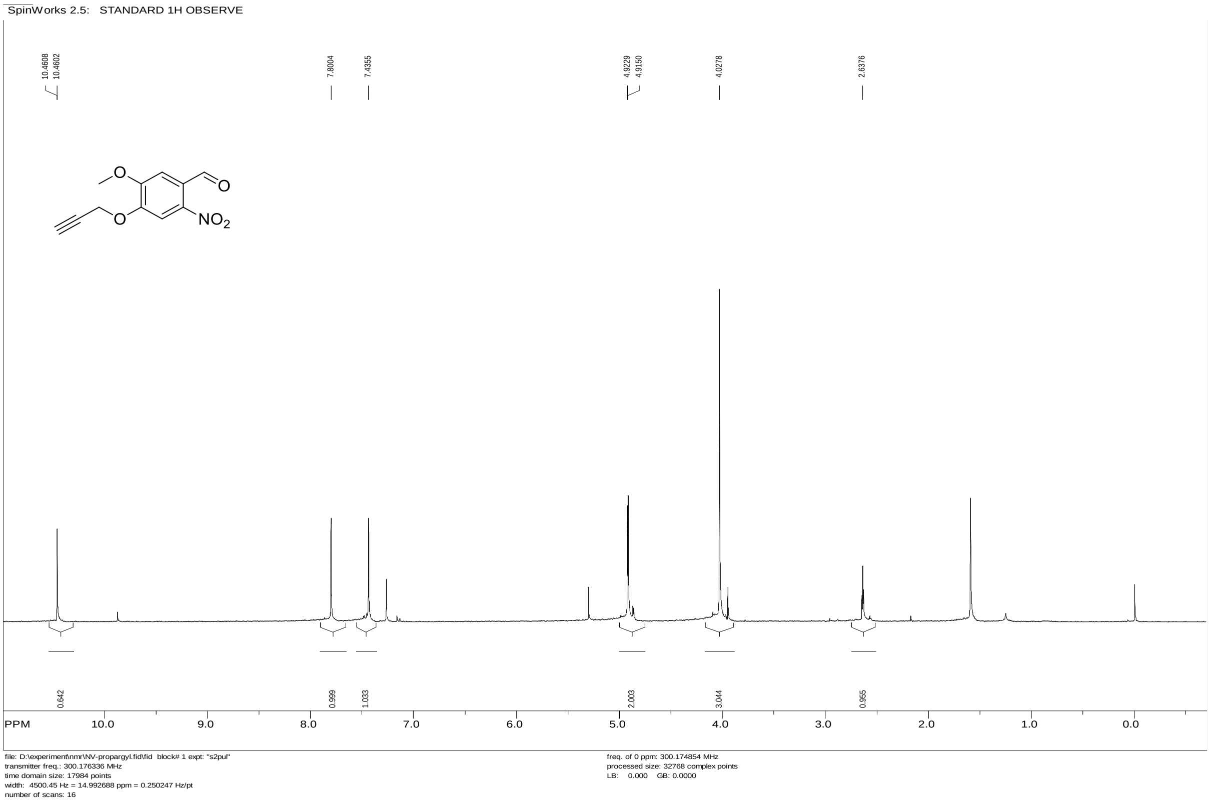

Figure S9. ^{1}H NMR spectrum of **5.**

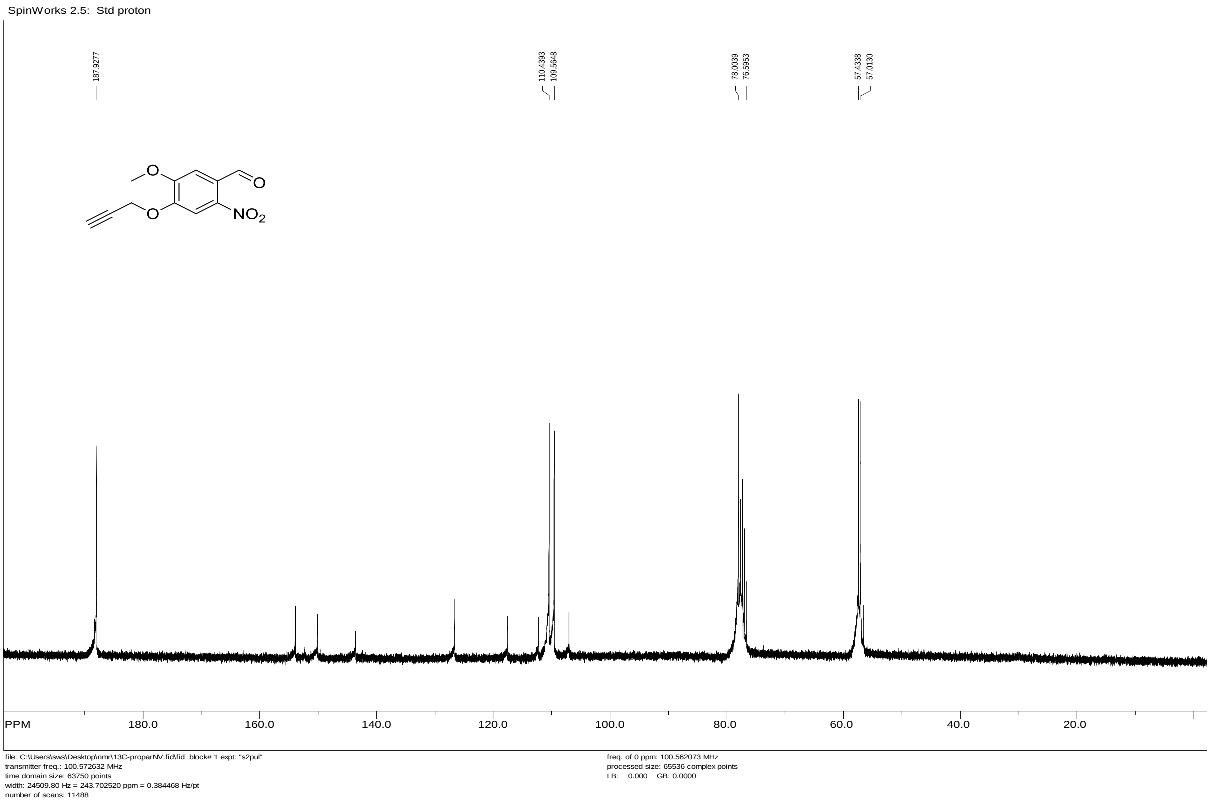

Figure S10. ^{13}C NMR spectrum of **5.**

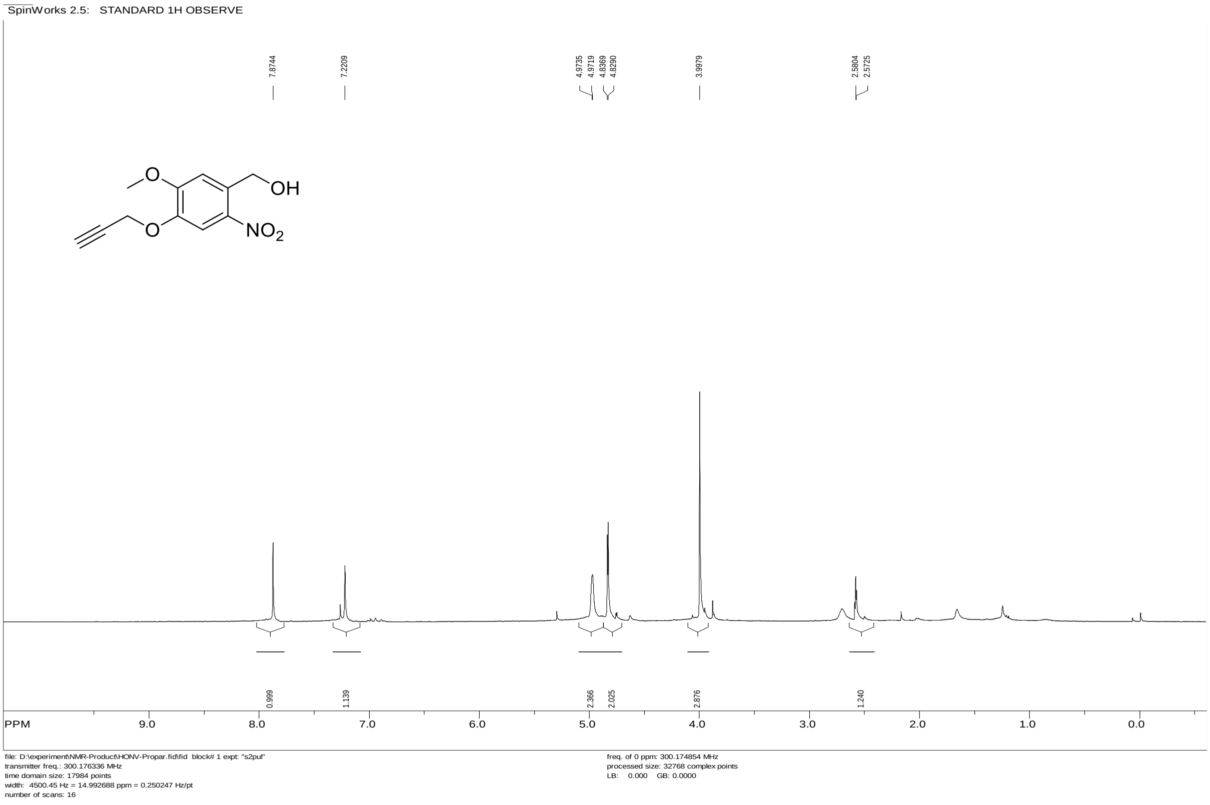

Figure S11. ^{1}H NMR spectrum of **6.**

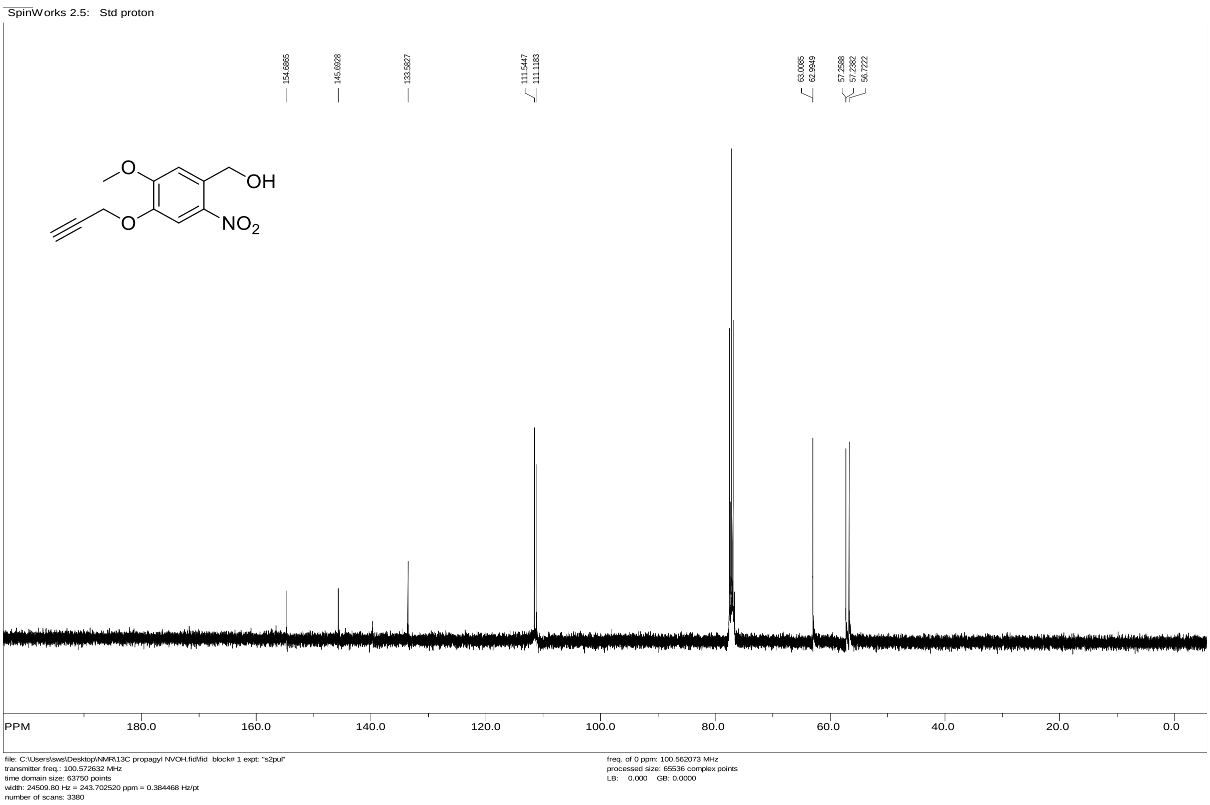

Figure S12. ^{13}C NMR spectrum of **6.**

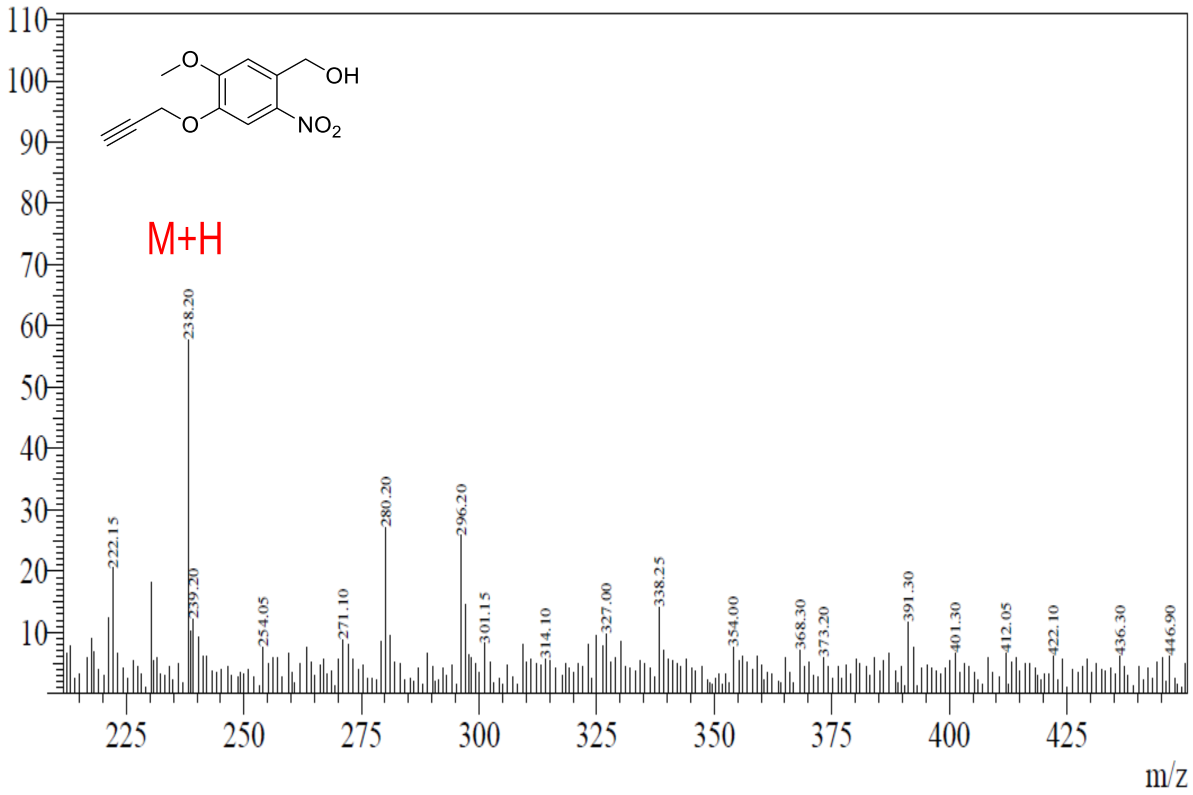

Figure 13. MASS spectrum of **6.**

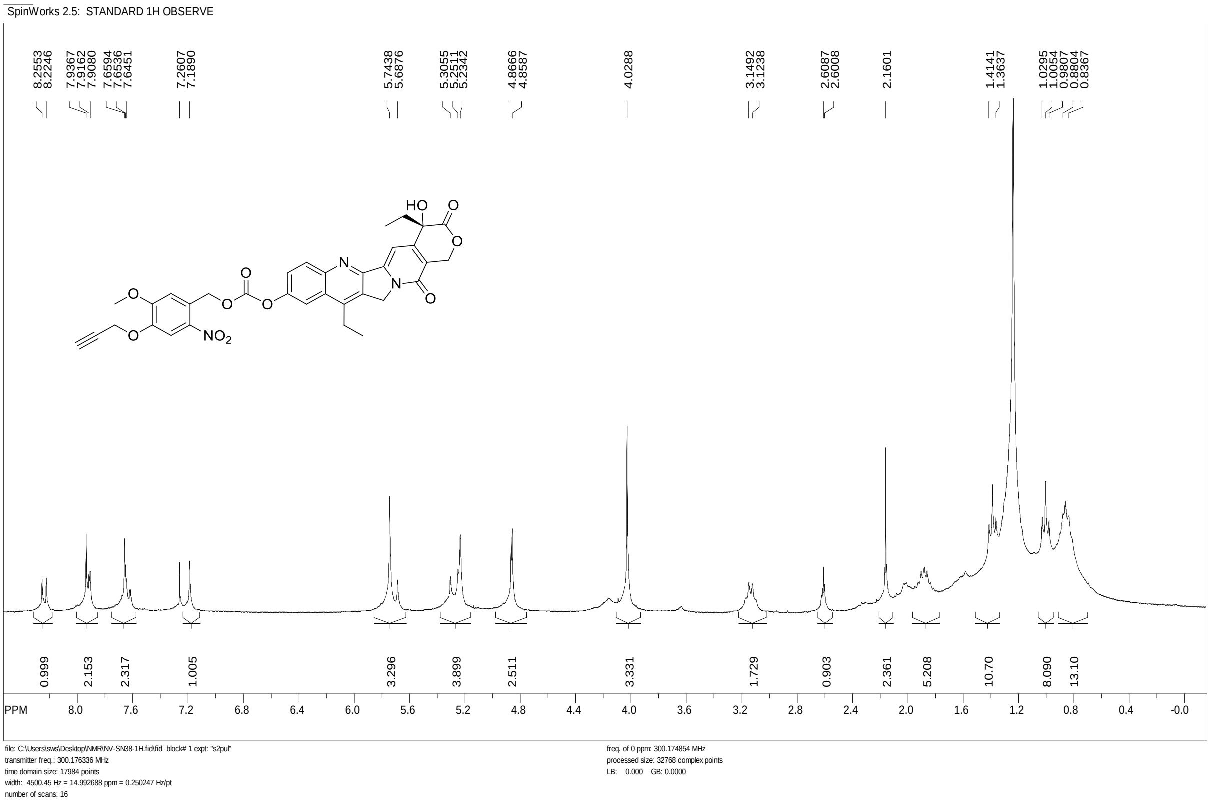

Figure S14. ^{1}H NMR spectrum of **7**

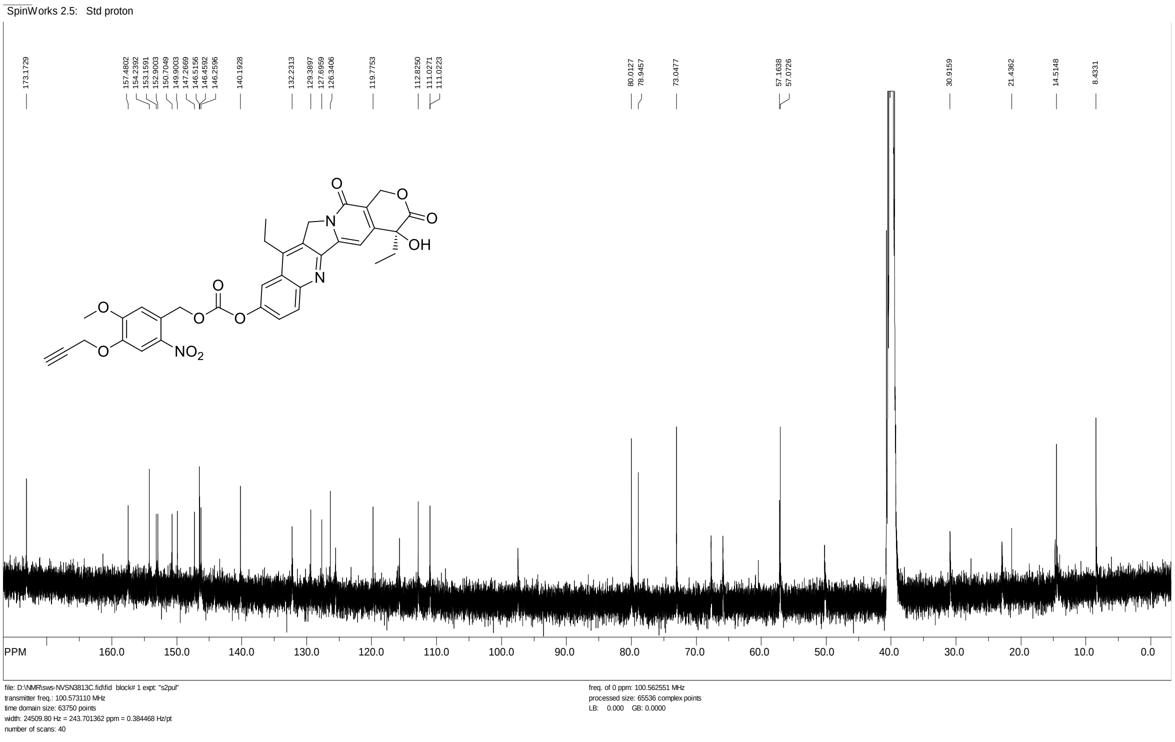

Figure S15. ^{13}C NMR spectrum of **7.**

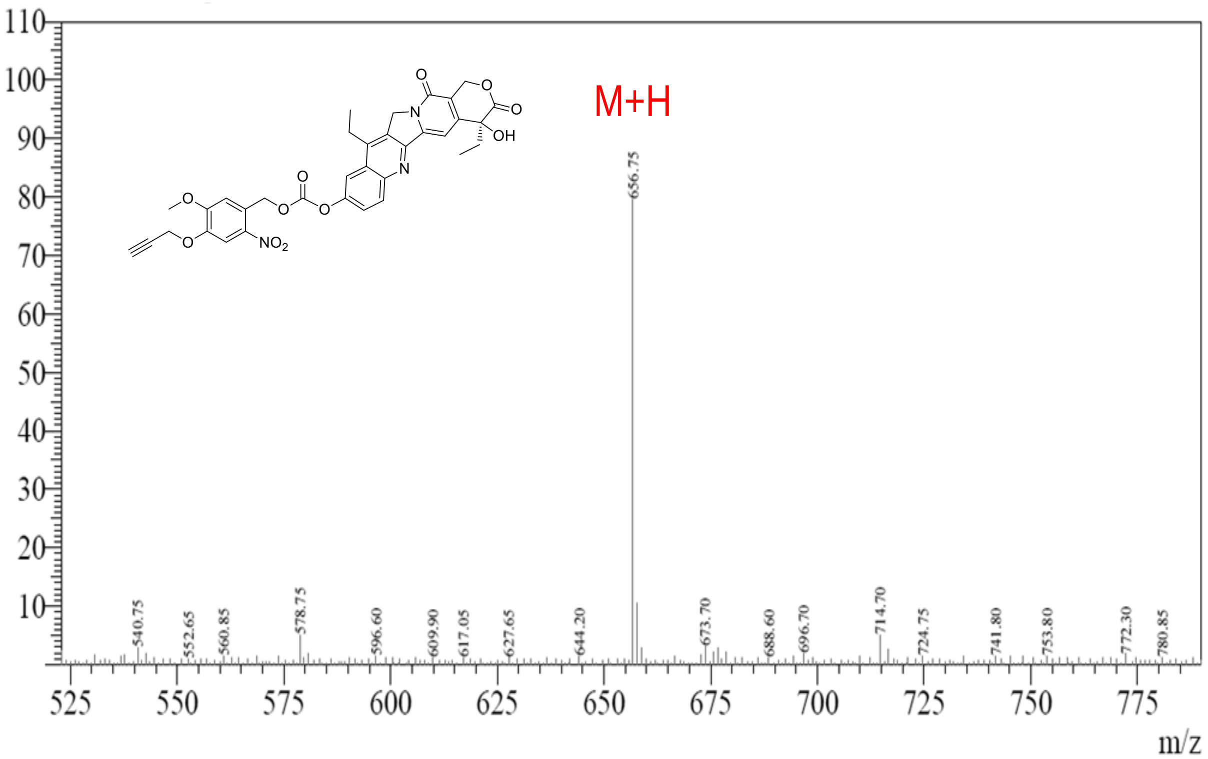

Figure S16. MASS spectrum of **7.**

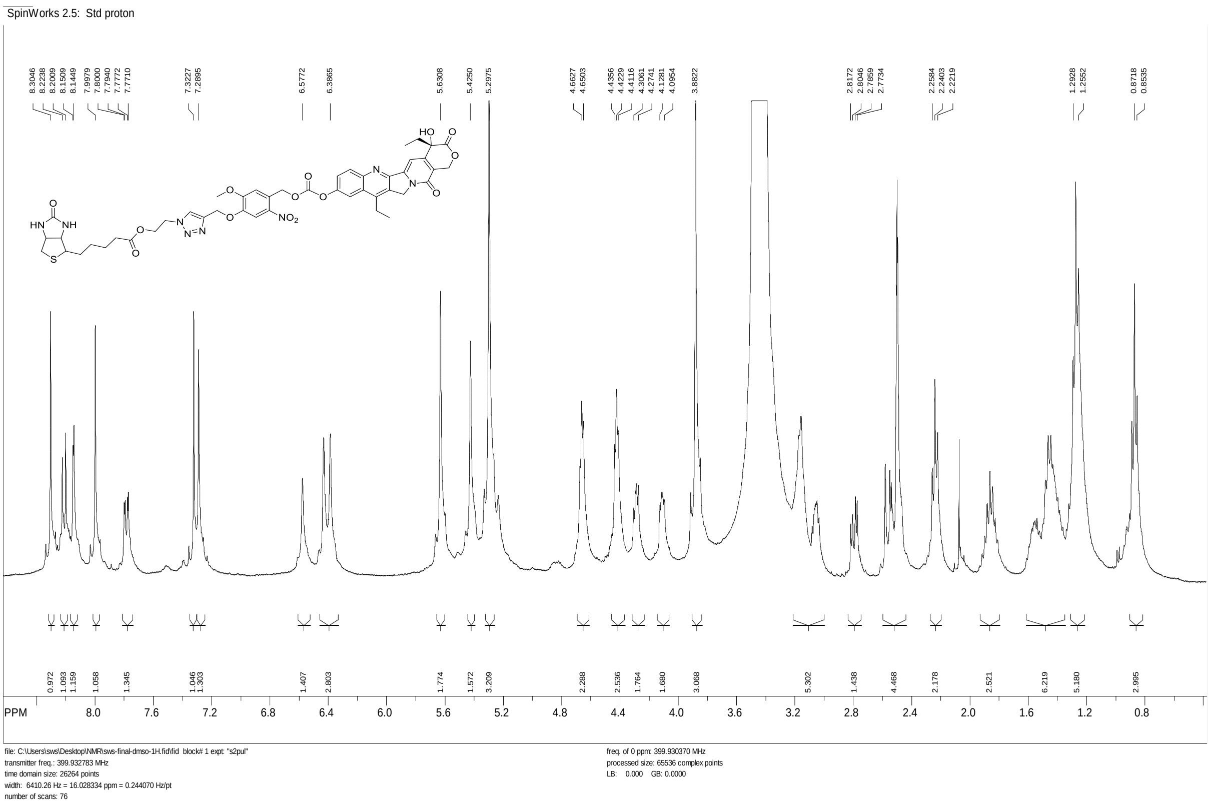

Figure S17. ^{1}H NMR spectrum of **1 (PT-1)** in DMSO-d_6**.**

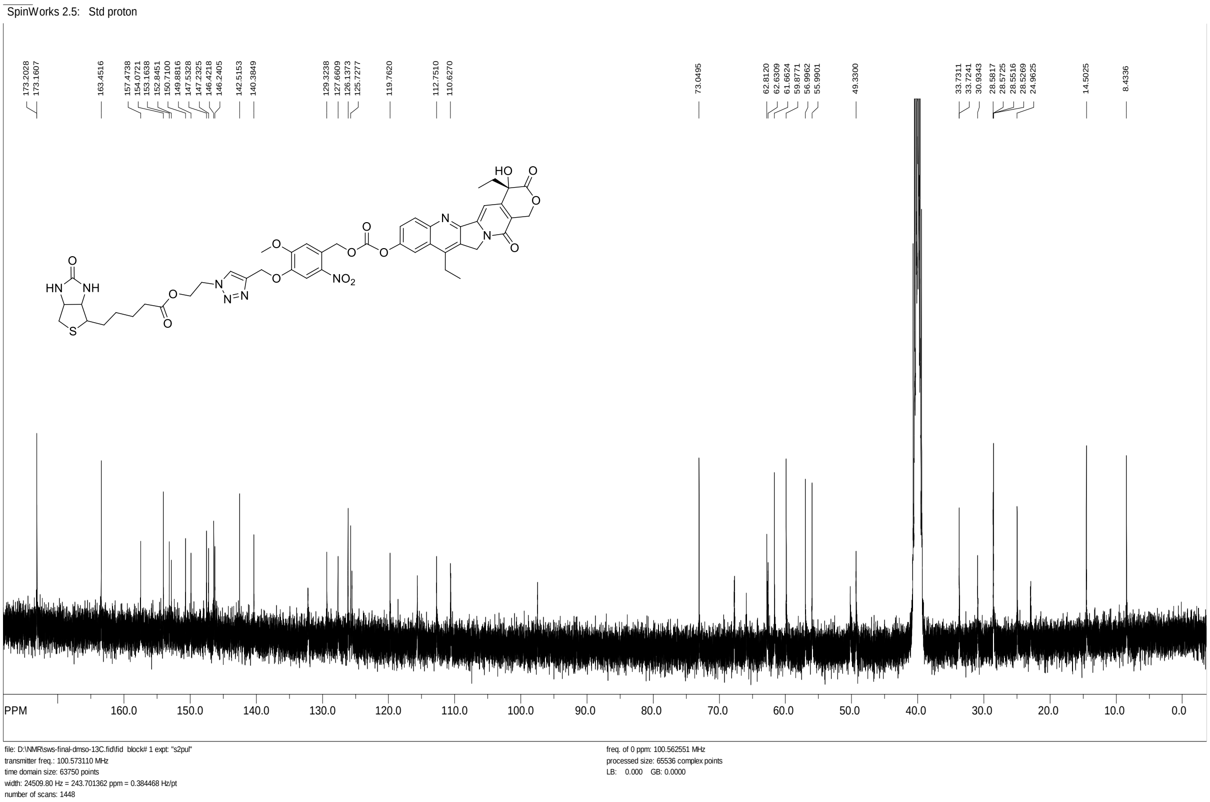

Figure S18. ^{13}C NMR spectrum of **1 (PT-1)** in DMSO-d_6.

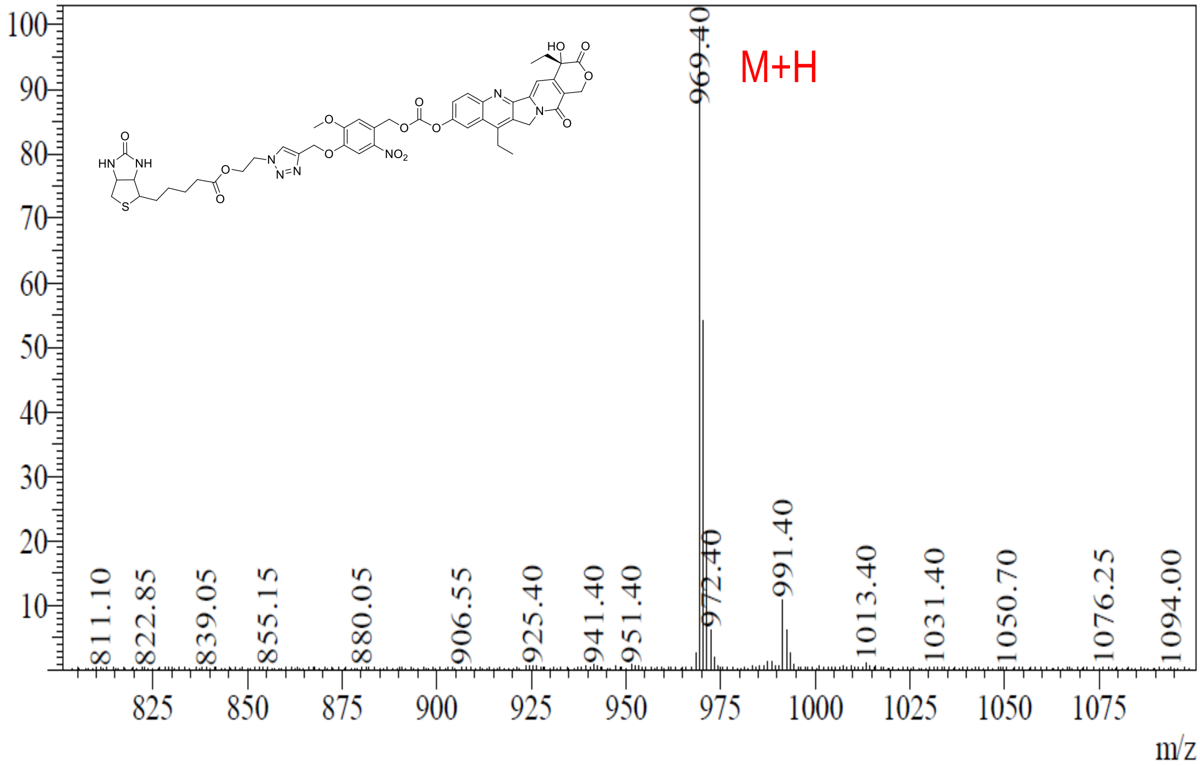

Figure S19. MS spectrum of **1 (PT-1).**

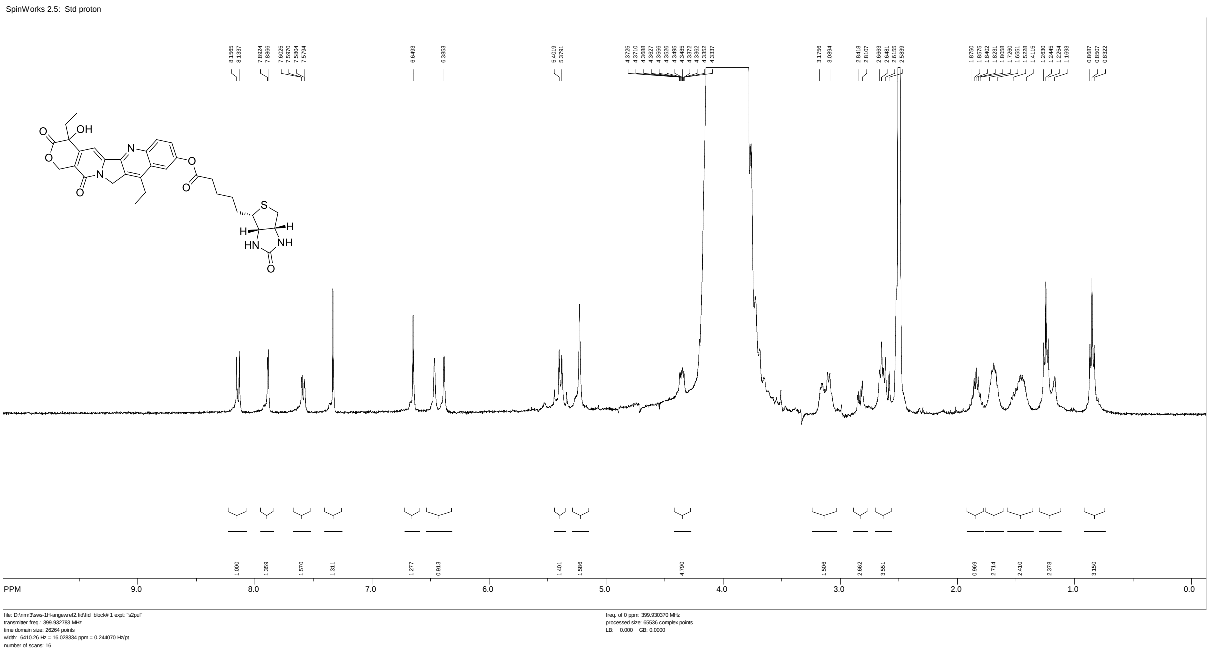

Figure S20. ^1H NMR spectrum of **8** (the broad ^1H peak at around 4 ppm is due to water contained in the DMSO-d_6 solvent used to record the spectrum).

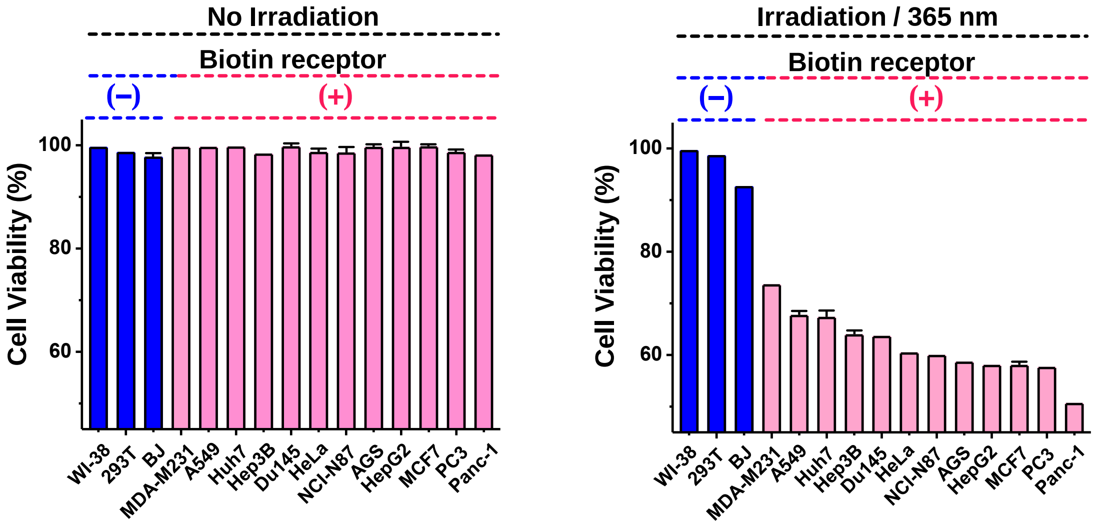

Figure S21. Cytotoxicity of **PT-1** in various cancer cell lines. Cells were treated with 10 nM **PT-1** for 30 min and irradiated with 405 nm laser light for 1 h. MTT assays were then performed.

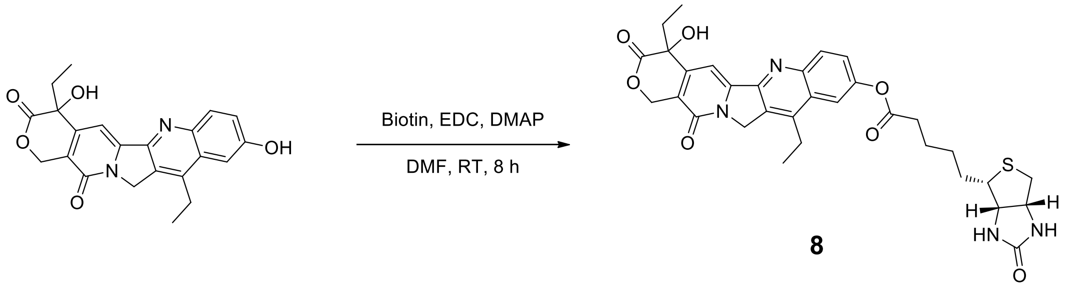

Figure 22. Synthesis of compound **8**.

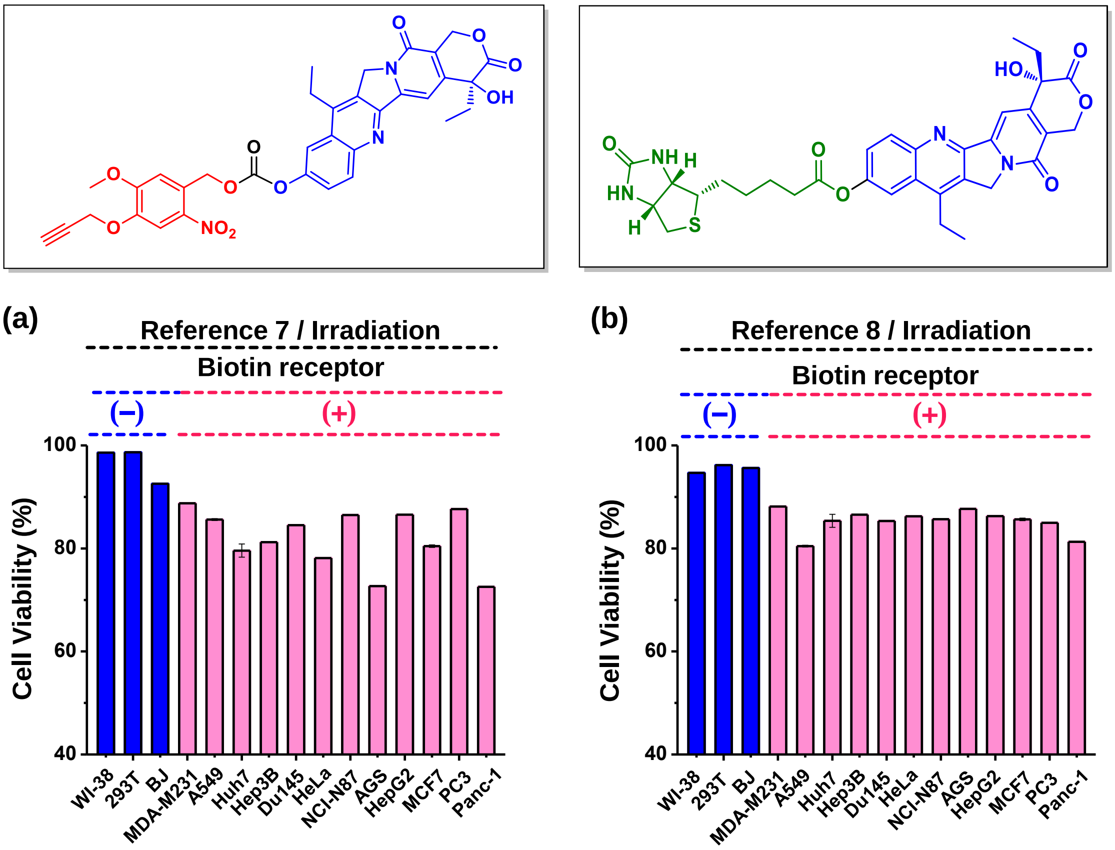

Figure S23. Cytotoxicity of Reference **7** (without biotin targeting unit) and Reference **8** (without photo-releasing unit) in various cancer cell lines. Note that cells were treated with 10 nM of the agent in question for 30 min and irradiated with 405 nm laser light for 1 h. Then MTT assays were performed.

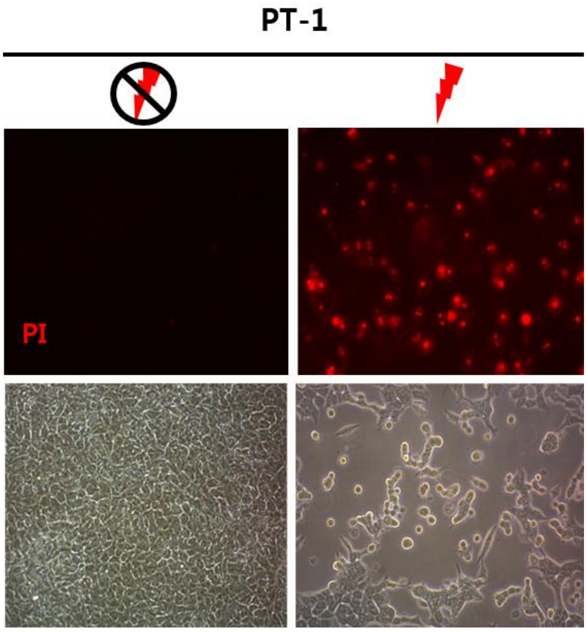

Figure S24. Fluorescent and phase contrast images of PI-stained A549 cells. The cells were treated with **PT-1** for 30 min and irradiated with 405 nm laser for 1 h. The images were taken 24 h after irradiation. Magnification: 200x.

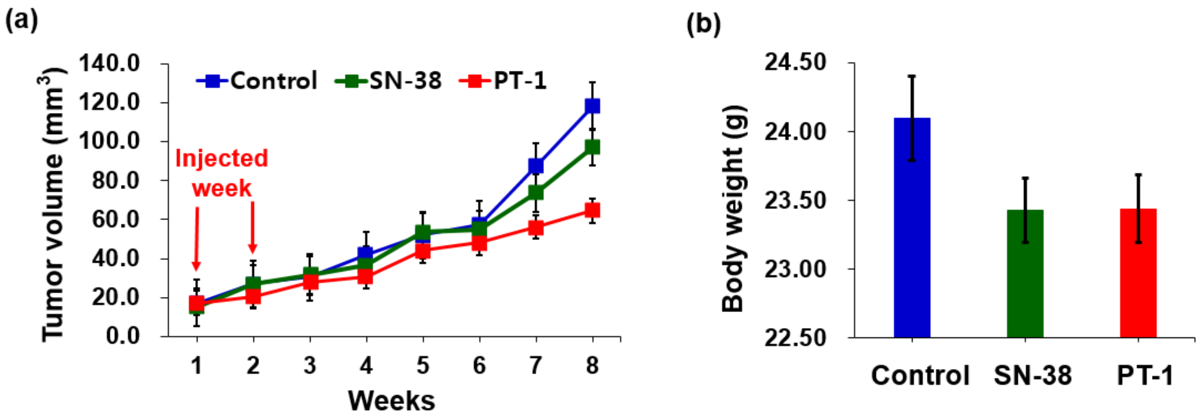

Figure S25. Quantitative analysis of tumor volume (a) and body weight (b) for three different treatment groups. Tumor volumes were calculated for tumor tissues irradiated with 405 nm laser light for 1 h; this was done 3 h post-injection. Mice were tail vein injected PBS, SN-38 (3 mg/kg), or **PT-1** (3 mg/kg for comparing to SN-38, whereas **PT-1** concentration for other *in vivo* study was 8 mg/kg). Body weights were measured at the end of the 8-week experiment (n = 3 per treatment).

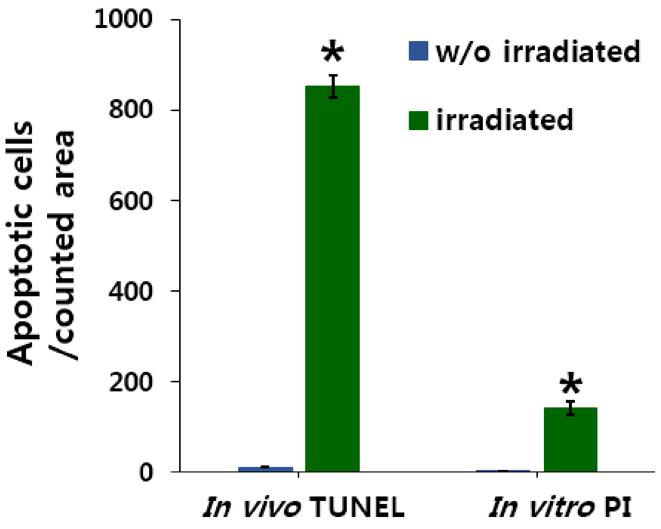

Figure S26. Quantitative analysis of apoptotic cells *in vivo* as monitored using tumor tissue sample and *in vitro* using A549 cancer cells. Tumor tissues were obtained from tumor-bearing mice, which were tail vein injected with **PT-1** (8 mg/kg, eight doses). A549 cells were treated with 10 nM **PT-1** for 1 h. TUNEL-and PI-positive cells were counted from 7 randomly selected areas that were either subject to photo-irradiation or not. Similar light and dark protocols were carried out in the case of the *in vitro* studies.

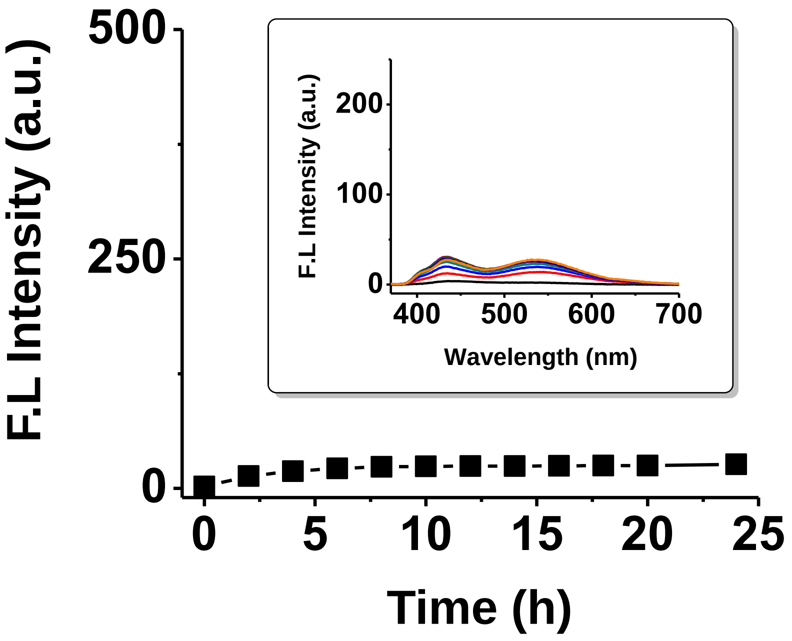

Figure S27. Time dependent changes in the fluorescence emission intensity of **PT-1** recorded in the absence of light in serum at 36 ˚C; λ_{ex} 365 nm; slit 3/3.

Table S1. Human cell lines used to determine IC_{50} values of **PT-1** after 30 min incubation and laser irradiation at 365 nm for 1 h.

Name of cell lines	Cell types	IC_{50} value (μM) of PT-1	Expression of biotin receptor[28]
**Normal cells**			
WI-38	Lung fibroblast	**>1**	**Na**
293T	Embryonic kidney	**>1**	**Na**
BJ	Foreskin fibroblast	**>1**	**Na**
**Cancerous cells**			
MDA-M231	Breast cancer	**>1**	++
MCF7	Breast cancer	**0.51 ± 0.16**	+++
A549	Lung cancer	**0.50 ± 0.86**	+++
HeLa	Cervical cancer	**0.80 ± 0.22**	+++
HepG2	Hepatic cancer	**0.87 ± 1.63**	+++
Huh7	Hepatic cancer	**0.85 ± 0.27**	+++
Hep3B	Hepatic cancer	**0.75 ± 0.24**	+++
NCI-N87	Stomach cancer	**0.65 ± 0.06**	+++
AGS	Stomach cancer	**0.63 ± 0.91**	+++
Du145	Prostate cancer	**0.53 ± 0.40**	+++
PC3	Prostate cancer	**0.53 ± 0.24**	+++
Panc-1	Pancreatic cancer	**0.11 ± 0.34**	+++

na: not applicable

Table S2. Primer information used in the RT-PCR analysis discussed in the main text.

Name of gene	Forward primer sequence	Reward primer sequence	Annealing Tm (°c) product size (bp)
TOP1	CAA GCA GCC CGA GGA TGA TC	GCA CTT TTC AGG TCT CTC CG	58 (333)
MRP1	GGC CTA ATG CCG AAC ACA TT	CAG CGT CTG GCC CTT CTTC	59 (149)
MRP2	ACA GAG GCT GGT GGC AAC C	GGG ACC CTG CGC ATG AAC CTG	56 (348)
BCRP	AGT TCC ATG GCA CTG GCC ATA	TCA GGT AGG CAA TTG TGA GG	59 (379)
FADD	GGA CCC GTT CCT GGT GCT GC	GTC TAG GCC GCT CTG CAC GC	60 (130)
TRAIL	GCG CAG CGA GTG GGA CAG AG	GGC ACT GGG TCC GTG CTG TC	60 (130)
FASL	GGA TTG GGC CTG GGG ATG TTT CA	TTG TGG CTC AGG GGC AGG TTG TTG	58 (334)
BID	AAC ACC AGC CGG TCG GAG GA	GGC GTG TGA CTG GCC ACC TT	60 (130)
BAK	CTG CAA CCT AGC AGC ACC AT	TGC TGG TAG ACG TGT AGG GC	55 (130)
CASPASE-3	TGG TGT TGA TGA TGA CAT GGC GTG	AGC ATG GCA CAA AGC GAC TGG A	60 (130)
CASPASE-9	TCG AGG ACA TCC AGC GGG CA	GCC AGC ATG TCC TGG CCT GT	60 (130)
GAPDH	TTC AGT GGT GGA CCT GAC CT	CAC CAC CCT GTT GCT GTA GC	60 (256)
